# Wild house mice have a more dynamic and aerotolerant gut microbiota than laboratory mice

**DOI:** 10.1186/s12866-025-03937-1

**Published:** 2025-04-09

**Authors:** Eveliina Hanski, Susan Joseph, Michael A. Curtis, James W. Swann, Marie Vallier, Miriam Linnenbrink, John F. Baines, Jens-Kjeld Jensen, Andrew Wolfenden, Iris Mair, Kathryn J. Else, Janette E. Bradley, Wieteke Holthuijzen, Jonathan H. Plissner, Aura Raulo, Maude Quicray, Sarah C. L. Knowles

**Affiliations:** 1 Department of Biology, University of Oxford, Oxford, UK; 2 Faculty of Medicine, University of Helsinki, Helsinki, Finland; 3 Centre for Host-Microbiome Interactions, King’s College London, London, UK; 4 Columbia Stem Cell Initiative, Columbia University, New York, USA; 5 Max Planck Institute for Evolutionary Biology, Plön, Germany; 6 Institute of Experimental Medicine, Kiel University, Kiel, Germany; 7 Í Geilini, Nólsoy, Faroe Islands; 8 School of Life Sciences, University of Nottingham, Nottingham, UK; 9 Lydia Becker Institute of Immunology and Inflammation, University of Manchester, Manchester, UK; 10 Institute of Ecology and Evolution, University of Edinburgh, Edinburgh, UK; 11 Department of Ecology & Evolutionary Biology, University of Tennessee, Knoxville, USA; 12 U.S. Fish and Wildlife Service, Midway Atoll NWR, Midway Island, USA; 13 Department of Computing, University of Turku, Turku, Finland; 14 Department of Functional and Evolutionary Entomology, University of Liège, Liège, Belgium

**Keywords:** Gut microbiota, House mice (*Mus musculus*), Wild vs lab, Bacterial aerotolerance, Microbiota turnover

## Abstract

The mammalian gut microbiota is a complex microbial community with diverse impacts on host biology. House mice (*Mus musculus*) are the major model organism for research on mammals, but laboratory domestication has altered their gut microbiota from that of their wild counterparts. Knowledge about how and why the gut microbiota of this species varies between lab and wild settings and among natural populations could improve its utility as a model organism. Here, we use a large dataset comprising over 800 house mouse samples from multiple laboratory facilities and strains and wild mice from mainland and island populations to investigate gut microbiota variation in this species across contrasting genetic and environmental settings. Across geographically disparate populations, we find that wild mice possess a gut microbiota that is compositionally distinct, displays a higher relative abundance and richness of aerotolerant taxa, and is taxonomically and functionally more diverse than that of lab mice. Longitudinally sampled wild mice also display markedly higher temporal turnover in microbiota composition than lab mice. Wild mice from oceanic islands harboured microbiotas that differed subtly from those of mainland wild mice and were more divergent from lab mouse microbiotas. These findings highlight much greater spatial and temporal turnover of gut microbes in wild compared to laboratory mice.

**Supplementary Information:**

The online version contains supplementary material available at 10.1186/s12866-025-03937-1.

## Introduction

The mammalian gut houses a diverse collection of microbial organisms known as the gut microbiota, that provides many important functions for the host. It is involved in several developmental processes, such as growth, immune maturation, and central nervous system development [1–3], but also in processes operating into adulthood such as immune regulation, metabolism, and protection against pathogens [4–6]. The laboratory mouse (*Mus musculus*) is the predominant model system for fundamental and biomedical research on mammals, providing a powerful system in which factors from host genotype to diet can be tightly controlled, biological processes can easily be studied across the lifespan, and the microbiota can be readily manipulated. However, laboratory mice live in an artificial world where individuals are typically inbred, housed under stable environmental conditions and exposed to a limited number of other individuals, raising concerns about the relevance of gut microbiota findings from laboratory mice, as well as the influence lab-adapted gut microbiotas on mouse research more broadly [7, 8].

Indeed, past studies have suggested laboratory mice harbour a different gut microbiota from that of their wild house mouse relatives [9–12] and results from recent microbiota transplant experiments indicated that a wild mouse-derived gut microbiota can induce quite different phenotypes to lab-mouse derived gut microbiotas, that may be more relevant for understanding the microbiota’s impact on human health [13]. However, with relatively few studies comparing lab and wild *Mus musculus* microbiotas to date, it remains unclear in which respects lab mouse microbiotas consistently differ from those of their wild counterparts, including in functional and phenotypic characteristics, as well as temporal stability. Considering how the gut microbiota varies across a wider range of genetic and environmental backgrounds in both lab and wild settings [14–17] is important to comprehensively understand the extent to which domestication has influenced the gut microbiota of the house mouse. Improved cataloguing of the house mouse gut microbiota across space and time is also important for developing a relevant range of natural microbiotas for use in wild-reconstituted lab model organisms [18] and knowledge of which common gut microbes should be included in mouse-specific synthetic communities.

Here, we perform a comparative analysis of among- and within-individual variation in the house mouse gut microbiota, across multiple wild and lab settings. We analyse over 850 faecal samples from lab-reared mice of multiple strains and from several animal facilities as well as from wild mice caught in various island and mainland locations across the globe, some sampled repeatedly over time. Considering that microbial exposure patterns vary significantly between lab and wild mice, we hypothesised that wild and laboratory mice would have distinct gut microbiotas, not just in terms of their compositional and taxonomic profiles but also in terms of their within-individual dynamics. Further, we hypothesised that if island-dwelling populations of mice are more isolated and subject to more variable selection pressures, they may possess more divergent, population-specific gut microbiotas than mainland populations.

## Methods

### Sample collection

We collected faecal samples from seven wild populations and six laboratory mouse colonies (groups of mice of the same genetic strain housed in the same facility) across three animal facilities in the UK (Biomedical Services Building, Oxford; Kennedy Institute, Oxford; King’s College London; Table S1). All lab mouse colonies were sampled between November 2020 and May 2021. Among the laboratory mouse colonies, three were C57BL/6 wild-type mice and three were transgenic; CCSP-rtTA, Pdgfra-creER, and SKG. The latter was sampled after intestinal inflammation was induced with curdlan injection, while no other sampled mice were subject to interventions. All laboratory mice sampled were adult (> 3 months of age) and reproductively inactive. To sample laboratory mice, faecal pellets were collected from mice placed on a sterile surface, immediately preserved in DNA/RNA Shield (Zymo Research, Irvine, California, USA) then stored at -80°C until DNA extraction.

All sampled wild mice were from *Mus musculus* populations from either Europe or the Hawaiian archipelago (Midway Atoll). The most heavily sampled population derived from Skokholm Island, an oceanic island off the coast of Wales, UK, where we sampled mice on five field trips between 2019 and 2021. This yielded a total of 948 samples from 337 unique mice (mean 2.5 samples per mouse, range 1–12; Table S1). Mouse trapping methods are detailed in Hanski et al. 2023 [19]. Briefly, small Sherman traps baited with 4 g peanuts and containing non-absorbent cotton wool as bedding were set at dusk and collected at dawn. Two sampling sites on the island were trapped, typically with three consecutive nights at one site before switching to the other over a period of several weeks. Traps showing any evidence of mouse contact were washed thoroughly and sterilised with 20% bleach solution before being re-used. Newly captured mice were uniquely identified either using a subcutaneous passive integrated transponder (PIT) tag or a unique ear clip ID if too small to be tagged. All captures were aged, sexed, measured for body length (from the nose tip to the base of the tail) and weighed, before release within 2 m of their trapping point. Pregnant, lactating, and perforate female mice as well as males with visibly descended testes were classified as reproductively active. Age classification (juvenile, sub-adult or adult) was based on body weight and reproductive state [20]: reproductively inactive mice weighing ≤ 15.0 g were classed as juveniles, mice ≥ 20.0 g were classed as adults regardless of reproductive state, and mice weighing between 15.1 and 20.0 g or under 15.0 g but reproductively active were classed as sub-adults. Faecal samples were collected from traps in a sterile manner and preserved in DNA/RNA Shield (Zymo Research, Irvine, California, USA). Samples were stored at -20^o^C while on the island (up to 6 weeks), after which samples were transported back to the lab and stored at -80^o^C (for up to 17 months) until DNA extraction.

Gut microbiota samples from the six other wild mouse populations were acquired through collaboration (Table S1). Because of this, sample types and storage methods varied somewhat across populations. Samples from Midway Atoll, the Faroe Islands, Cologne and Espelette were intestinal contents taken from trapped and dissected animals, and samples from the Isle of May and Oxford were faecal pellets. Further, while samples from Skokholm, Wytham, and all laboratory colonies were preserved in DNA/RNA Shield, samples from other wild mice had been preserved differently: Midway Atoll and Faroe Islands samples had been stored at -20^o^C or -80^o^C in isopropyl alcohol (Midway Atoll) or without preservative (Faroe Islands), but were transferred to tubes containing DNA/RNA Shield before shipping to the UK. Samples from the Isle of May (collected fresh at the time of handling [21]) had been stored at -80^o^C without preservative and were shipped to Oxford on dry ice. Samples from Cologne [22] and Espelette [23, 24] were dissected and intestinal content was stored in RNAlater and PBS, respectively, and shipped to Oxford on dry ice. Mice from which non-faecal samples were used as proxies of microbiota composition were euthanised using either cervical dislocation (Midway Atoll) or rising levels of CO_2_ (Cologne and Espelette).

Although others have demonstrated limited differences between large intestinal and faecal microbiota compositions in mice [25–28], we tested the impact of variable preservative methods on microbiota composition using a subset of 15 mouse faecal samples (from different individuals) sampled on Skokholm Island. For these samples, replicate aliquots were stored in either DNA/RNA Shield, RNAlater, absolute ethanol, or without preservative, and stored and transported as described above. All four replicates from a given sample were DNA extracted in the same extraction batch, and processed in a single round of library preparation and amplicon sequencing.

### DNA extraction, library preparation and sequencing

DNA was extracted from all samples using ZymoBIOMICS DNA MiniPrep kits, according to manufacturer’s instructions for use on faecal samples (Zymo Research, USA). Samples were randomised into 64 extraction batches of up to 23 samples. A negative extraction control (40µL of DNAse-free H_2_O) was included in every extraction batch except one, in variable tube positions. For samples preserved in DNA/RNA Shield, this preservative was used as a lysis solution in the first step of DNA extraction. For other samples, samples were centrifuged and the preservative removed by pipetting, after which ZymoBIOMICS Lysis Solution was added in the first step of DNA extraction. Library preparation and amplicon sequencing was completed by the Integrated Microbiome Resource (IMR), Dalhousie University, using the protocol described in Comeau et al. (2017) [29]. Briefly, the V4–V5 region of the bacterial 16 S rRNA gene was targeted using primers 515(F) and 926(R) [30, 31]. Amplification was conducted in 16 library preparation plates each containing up to 95 samples plus a negative PCR control, followed by sequencing in 5 runs using the Illumina MiSeq platform (Reagent kit v3, 2 × 300 bp chemistry). All extraction controls (*n* = 63) and PCR controls (*n* = 16) were sequenced.

### Microbiota data processing

Microbiota data were processed and analysed in R version 4.1.2 [32]. Sequences were denoised, chimeras removed, and amplicon sequence variants (ASVs) inferred using *DADA2* version 1.16 [33], with the pipeline run separately for each sequencing batch. Taxonomy was assigned using the SILVA rRNA database version 138. Further processing was conducted separately for the wild and lab mouse datasets. Contamination was tested for by assessing reads in negative controls. Among all controls except one (63 extraction controls and 16 PCR controls), few reads were detected in negative controls, with a mean of 75 reads (median 13, range 0 to 1,394). In one PCR control ASV diversity and read count were much higher (255 ASVs and 29,300 reads), more similar to biological samples (which had a mean of 29,235 reads). All 5 extraction controls from the same 96-well plate that contained this apparently contaminated PCR control (*n* = 5) contained very few reads (< 20 each), indicating the entire plate was not contaminated during library preparation. Rather, the most likely explanation is that a biological sample was mistakenly pipetted into the control well in addition to its designated well. Since all other controls on this plate were negative, we retained this plate in our analyses. R package *decontam* [34] was used to identify potential contaminants, using the ‘prevalence’ method with default parameters. This identified 31 contaminants which were removed from the dataset. ASVs assigned as chloroplast or mitochondrial sequences were also removed. R packages *DECIPHER* and *phangorn* were used to build a microbial phylogenetic tree, and package *iNEXT* [35, 36] was used to generate separate sample completeness and rarefaction curves for lab and wild mouse datasets. Based on these curves, wild and lab mouse samples with a read depth below 5000 and 7,500, respectively, (where the curves plateaued) were excluded. Data were not rarefied [37]. The mean read count for samples included in the analyses was 29,815 (range 5,312–171,337) for wild mice and 25,588 (8,251–60,892) for lab mice. Asymptotic ASV richness and Shannon diversity were then estimated in *iNEXT*. Prior to beta diversity analyses, singleton and doubleton ASVs were identified and removed separately in the wild and lab mouse datasets. ASV counts were normalised to relative abundance and beta diversity metrics (Jaccard distance (binary), Aitchison distance, weighted and unweighted UniFrac distances) were calculated across sample pairs in R package *phyloseq* [38]. A centred log-ratio (clr) transformation was performed when calculating Aitchison distance, using the R package *microbiome* [39]. Here, zero relative abundance values were replaced with a pseudocount as follows: min(relative abundance/2).

### Functional profile characterisation

Functional pathways were predicted from the 16 S rRNA data using Phylogenetic Investigation of Communities by Reconstruction of Unobserved States 2 (PICRUSt2) version 2.5.0 pipeline (picrust2_pipeline.py) using default options [40] and the MetaCyc Metabolic database. Number of unique pathways in each sample was then determined.

### Classification of microbial aerotolerance

Bacterial aerotolerance information was retrieved from Bergey’s Manual of Systematics of Archaea and Bacteria, with additional peer-reviewed publications consulted when aerotolerance data was not present in the manual. Aerotolerance was assigned based on genus-level taxonomy, unless (1) aerotolerance information was not available or (2) genus-level taxonomy was not assigned, in which case family-level aerotolerance information was sought from Bergey’s manual or other references if required, and used if all genera within a family were stated in Bergey’s manual to have the same aerotolerance classification. Bacteria were categorised into one of three aerotolerance groups: *obligate anaerobes* (when specifically stated to be *obligately* anaerobic), *aerotolerant* (anything other than obligate anaerobes for which aerotolerance information was available, including facultative anaerobes), or *unknown* (where aerotolerance was unknown for a given genus). If a given genus included both obligate anaerobes and aerotolerant taxa, a Nucleotide BLAST search was conducted for the associated 16 S rRNA-derived ASVs. If a 100% species identity match was found with 100% query cover (~ 370 bp query length) and 0.0 E-value, the aerotolerance category was assigned based on this species.

## Statistical analyses

### Analysis of preservative effects on microbiota diversity and composition

To assess potential effects of sample storage system (DNA/RNA Shield, RNAlater, absolute ethanol, no preservative) on alpha diversity, we fitted Bayesian regression models (brm, likelihood family of *beta*) with asymptotic ASV richness or Shannon diversity as response variables. Predictor variables included preservative, read count, and animal ID (random effect). Alpha diversity estimates and read count were scaled to 0–1 for interpretability. All models used default uninformative priors, and model performance was evaluated by ensuring Rhat values < 1.05, bulk effective sample sizes > 10% of posterior draws, and no excess divergent transitions. Posterior predictive checks confirmed that simulated posterior distributions closely matched observed data.

To further assess the impact of sample preservative on microbiota composition, we used principal coordinates analysis (PCoA) and marginal permutational multivariate analyses of variance (PERMANOVA) on Euclidean distances on centred log-ratio (CLR) transformed data (Aitchison distance) using the adonis2 function in R package *vegan* [41]. Preservative, read count and animal ID were included as predictors. Beta dispersion was tested for using the betadisper function in package *vegan*.

### Cross-sectional analyses of wild and lab mouse microbiota

For cross-sectional microbiota comparisons of wild and lab mice, we selected a subset of wild mice that were as comparable as possible to their lab counterparts. These mice were therefore, when information was available, limited to adults that were reproductively inactive and for those with known sampling date, limited to those collected in a short time period (September-November) to minimise the impact of possible seasonal microbiota change [28, 42]. Differences in alpha diversity and within-population beta diversity between wild and lab mice were tested for using Wilcoxon rank sum tests. These tests were permutational (with 1,000 permutations) in tests of beta diversity differences as beta diversity metrics are non-independent. To assess differences in mean microbiota composition between lab and wild mice, we used PCoA and marginal PERMANOVA on beta diversity metrics using the adonis2 function in R package *vegan* [41]. Beta dispersion was tested for using the betadisper function in package *vegan*. Random Forest regressions (ntree = 10000, importance = TRUE, default mtry) in R package *randomForest* [43] were also used to identify key taxa driving differences in the gut microbiota of wild and laboratory mice, as well as mainland and island mice, with mean decrease in Gini used as a measure of importance.

### Longitudinal analysis of microbiota turnover in wild and lab mice

We used data from one wild population (Skokholm Island) and one laboratory colony (C57BL/6 in Facility B) where mice were repeat-sampled to compare within-individual microbiota turnover using Jaccard distance in wild and lab mice. Here, we included all reproductively inactive Skokholm mice sampled more than once, without exclusion based on age or sampling season. For lab mice, we used data from one repeat-sampled colony of and included only reproductively inactive individuals (lab mice were housed in same-sex cages only). We examined how beta diversity among samples from the same individual varied as a function of the time between sampling points, modelled using either a (log)-linear model, or a quadratic plateau model in R package *easynls* [44] (best fit was assessed from AIC values). When assessing the effect of cage density, we focused on mice housed at densities of 3 (*n* = 9) or 5 (*n* = 5) per cage, as pair-housed mice (*n* = 2) were only sampled over short intervals (< 50 days), limiting temporal comparisons. As a complimentary approach, we selected those wild and lab mice with highly comparable sampling histories, that had been sampled 4–5 times at approximately one-week intervals. To maximise sample size, here we did not exclude mice based on reproductive state. We then compared in the wild and lab mouse subsets, how the representation of newly detected ASVs vs. those persisting from previous time-points, changed across time-points.

### Analyses of aerotolerance variation in lab and wild

We repeated the above-described analyses on microbial richness and beta diversity for anaerobic and aerotolerant subsets of the gut microbiota. To test whether the proportion of aerotolerant taxa out of taxa with known aerotolerance varies statistically between lab and wild mice, we used the function brm from R package *brms* [45] to fit a Bayesian regression model (likelihood family of beta) with proportion of aerobes out of taxa with known aerotolerance as response variable and source (lab/wild) as predictor. Colony/population ID was included as a fixed effect.

As we saw differences in ASV richness of aerotolerant taxa as well as the proportion of aerotolerant taxa, we examined whether sample collection methods in the wild could be driving this. For this, we used data from the Skokholm wild mouse population. We investigated whether faecal deposition’s exposure time to oxygen increases (1) the ratio between aerobes and anaerobes and (2) ASV richness of either aerotolerant or anaerobic taxa. We used brm models (likelihood family of beta) with either proportion of aerotolerant taxa out of taxa with known aerotolerance, ASV count of aerotoletant taxa or ASV count of anaerobic taxa as response variable and ‘maximum exposure time’ (measured from sample collection time; trapping was conducted overnight) as a predictor. Some faecal samples were kept cold after the animal had been removed from the trap but before the sample was collected and stored in DNA/RNA Shield, and this variable was included as a binary predictor (yes/no cold storage).

All brm models included read count as a fixed effect and Animal ID as a random factor. Models with proportion of aerotolerant taxa out of taxa with known aerotolerance response variable also included relative abundance of taxa with unknown aerotolerance as a fixed effect. All brm models used default (uninformative) priors. For all brm models, model performance was confirmed by ensuring that Rhat values were < 1.05, bulk effective sample sizes were > 10% of posterior draws and excess divergent transitions (> 10) after warm-up were avoided (for this, adapt_delta was set to 0.9 and max_treedepth to 12). Additionally, posterior predictive checks were conducted to evaluate model fit, confirming that the simulated posterior distributions aligned closely with the observed data, with only minor deviations in some areas.

### Comparison of Island and Mainland wild mouse microbiota

We used a marginal PERMANOVA to test whether mean microbiota composition differed between mainland and island-dwelling lab mice, and permutational Wilcoxon rank sum tests to test whether mean within- and between- population beta diversity, as well as mean microbiota distance to lab mice (Jaccard distance) differed between island-dwelling and mainland wild mice. To ensure comparability across populations, only samples from adult, non-reproductively active mice were used in these analyses, sampled in a limited time window (September and November) from Skokholm where sampling was more continuous than other island populations.

## Results

We analysed gut microbiota composition across mice from six laboratory colonies and seven wild populations, encompassing over 850 faecal samples across diverse locations, strains, and animal facilities. Given the large-scale and multi-source nature of this dataset, sample collection methods varied somewhat (Table S1). However, analysis of replicate samples from the same individual wild mice stored in alternative preservatives showed only a limited impact of preservative on microbiota richness and composition, which were much more strongly shaped by animal ID. Alpha diversity varied between individuals but was not influenced by storage system (Figure S1A). Similarly, microbiota composition was primarily shaped by individual identity, with animal ID explaining 75.7% of variation (PERMANOVA on Aitchison distance, *R*^*2*^ = 0.757, *p* = 0.001; beta dispersion, *F* = 2.405, *p* = 0.011), compared to only 2.1% variation explained by preservative in the same analysis (*R*^*2*^ = 0.021, *F* = 1.081, *p* = 0.028; beta dispersion, *F* = 0.149, *p* = 0.940; Fig. S1B). We thus expect differences arising from variation in preservative to be relatively small.

### Wild mice have compositionally distinct and taxonomically and functionally more diverse gut microbiota than laboratory mice

**Fig. 1:**
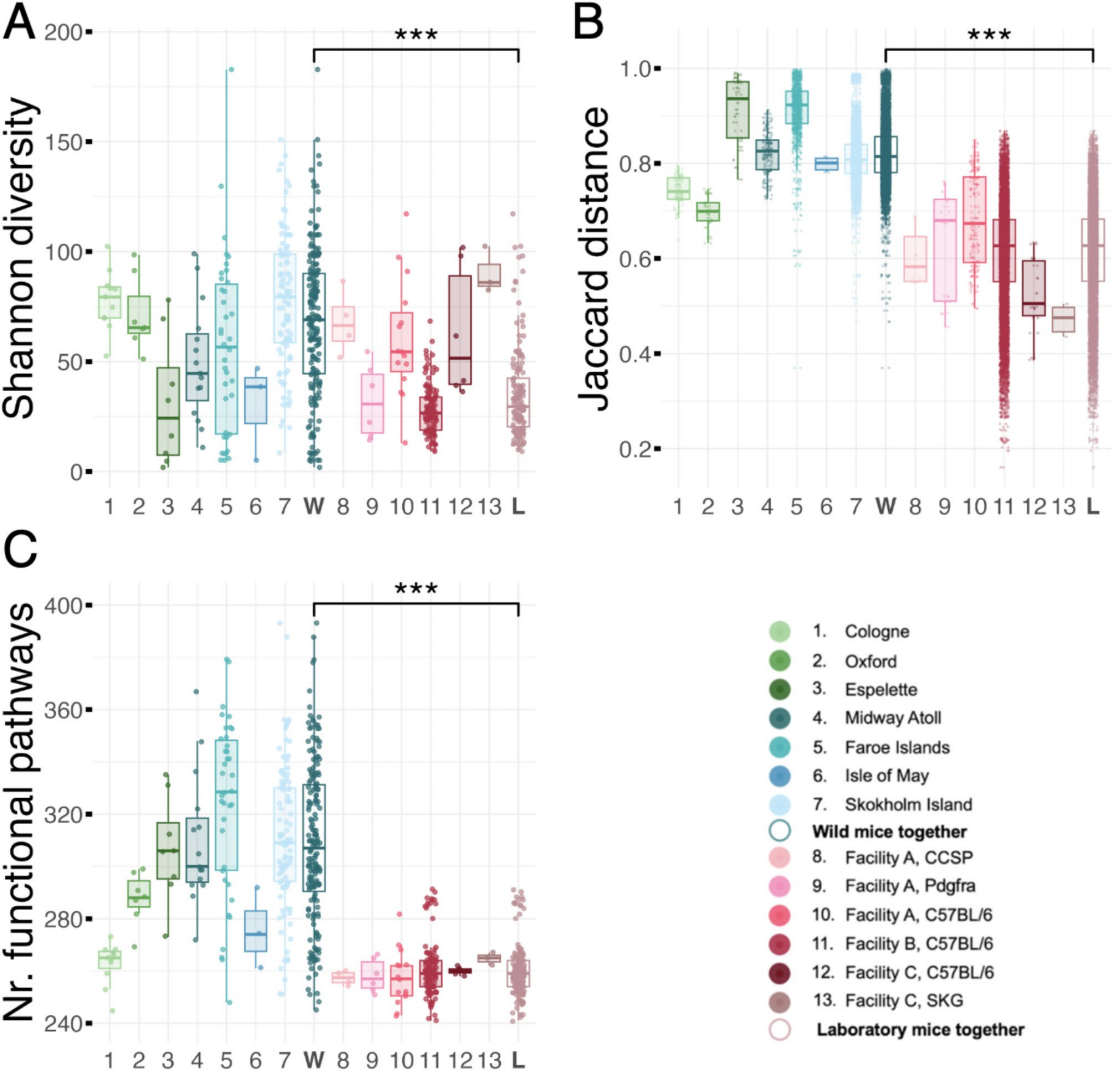
(**A**) Asymptotic Shannon diversity, (**B**) pairwise, within-population/colony Jaccard distance, and (**C**) number of unique functional pathways per mouse in wild (*n* = 180) and laboratory (*n* = 146) mice from seven populations and six colonies, respectively. Samples are from wild mice estimated to be adults based on body size, and lab mice over 3-months old. Boxplots are for individual wild mouse populations (*green =* mainland populations, *blue =* island populations) or laboratory mouse colonies (*pink*). Empty boxes represent all wild (*teal*, ‘W’) or laboratory (*pink*, ‘L’) mouse samples pooled. Statistical differences between wild and lab mice were tested with Wilcoxon rank sum tests (1,000 permutations used in dissimilarity tests; ***; *p* < 0.001)

Wild mice had significantly higher alpha diversity than lab mice, both in terms of ASV richness and Shannon diversity (Wilcoxon rank sum tests; both ASV richness and Shannon diversity *p* < 0.001; wild, *n* = 180; lab, *n* = 146). However, alpha diversity varied greatly between colonies and populations such that it was not consistently higher in every lab–wild comparison (Fig. 1A, Fig. S2).

The gut microbiota within wild populations was also more compositionally variable than within lab populations (Jaccard distance, permutational Wilcoxon rank sum test for lab vs. wild *p* < 0.001; wild, *n* = 180; lab, *n* = 146; Fig. 1B). On average, a pair of wild mice from the same population shared ~ 18% (standard deviation (sd) ± 6.5) of their combined ASVs. In comparison, lab mice shared approximately 39%, with this being the same value whether the pair came from the same colony (same strain and same facility; sd ± 11.5) or from the same animal facility (regardless of whether they were the same or different strain; sd ± 11.9). Across 1,928 ASVs detected in lab mice (*n* = 146), only 16 were detectable in > 90% of lab samples. 11 of these belonged to the family Muribaculaceae (genus *Muribaculum* or unknown) while others belonged to the genera *Colidextribacter*,* Ligilactobacillus*,* Desulfovibrio*,* Bacteroides*, and *Akkermansia.* In wild mice (*n* = 180), only 1 ASV among the 6,818 detected was found in > 90% samples, which was the identical *Ligilactobacillus* ASV detected in > 90% lab mouse samples. In a random subset of 100 wild and 100 lab mouse samples, a total of 5,735 unique ASVs were detected, with 73% only found in wild mice, 15% only in lab mice, and 12% in both systems; Fig. S3).

The wild mouse gut microbiota was also more functionally diverse, with more unique predicted functional pathways per individual than lab mice (Wilcoxon rank sum test, *p* < 0.001; lab, *n* = 146; wild, *n* = 180; Fig. 1C). Of the 415 unique functional pathways detected overall, all were found in wild mice but only 75% (312) in lab mice.

**Fig. 2:**
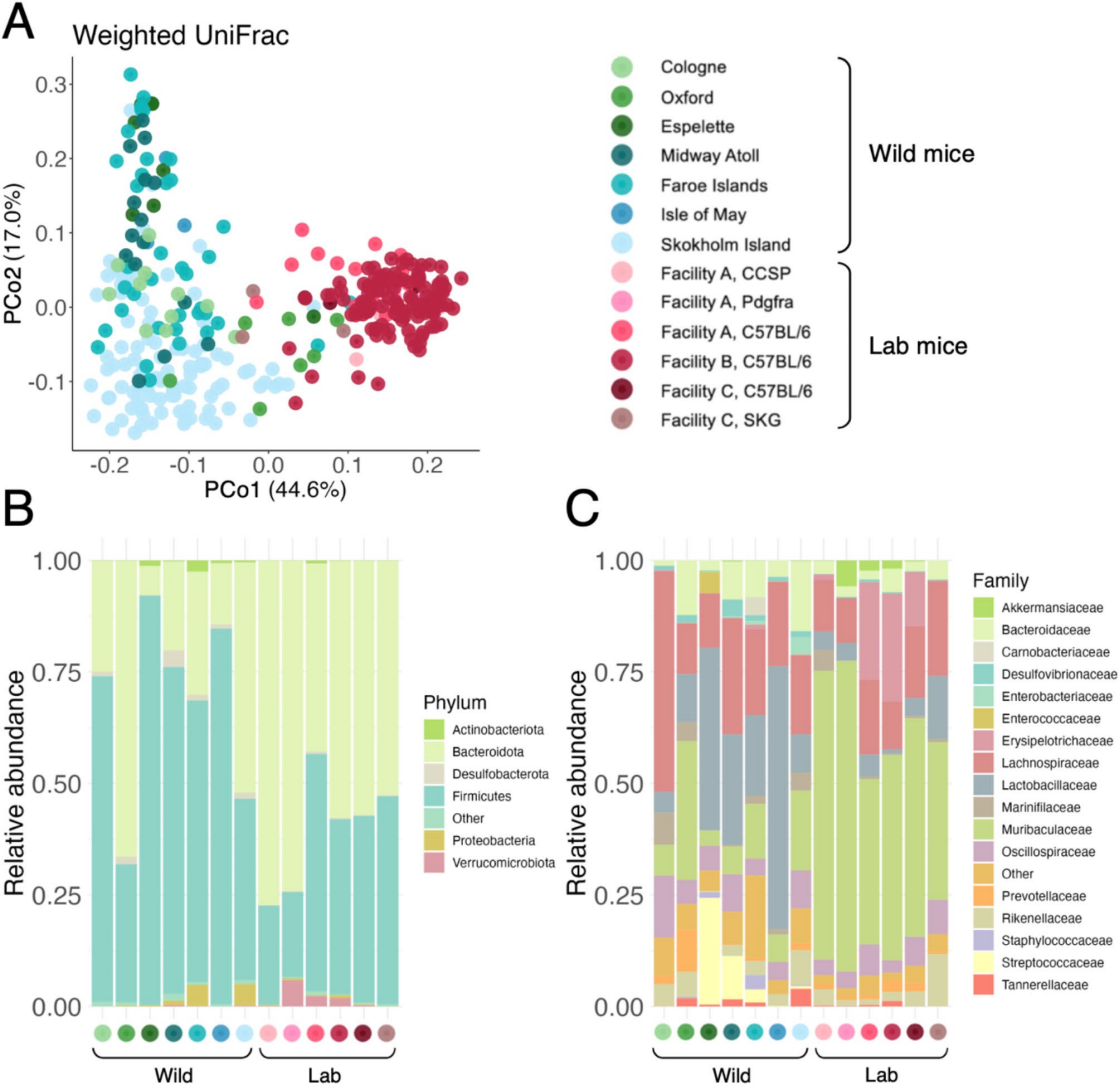
(**A**) Principal coordinate analysis of wild (*n* = 180) and laboratory (*n* = 146) mouse samples from seven populations and six colonies, respectively, on weighted UniFrac distance. Circles are individual samples coloured by population/colony (*green* = mainland wild mice, *blue* = island wild mice, *pink* = laboratory mice). (**B**–**C**) Mean relative abundance of bacterial (**B**) phyla and (**C**) families across seven wild populations and six laboratory colonies indicated by coloured circles on x-axis

The gut microbiota of wild and lab mice was also compositionally distinct, with samples clustering primarily by source (lab/wild) in principle coordinates analysis irrespective of the beta diversity metric used, and to a lesser extent by population (Fig. 2A, Fig. S4). Both strain and animal facility predicted a substantial proportion of variation in lab mice (univariate marginal PERMANOVAs with Aitchison distance: *strain*: *R*^*2*^ = 0.134, *F* = 7.367, *p* = 0.001; beta dispersion *F* = 1.123, *p* = 0.294; *facility*: *R*^*2*^ = 0.202, *F* = 18.180, *p* = 0.001; beta dispersion *F* = 91.143, *p* = 0.001; *n* = 146). Similarly, population ID explained ~ 12% of gut microbial variation among wild mice (PERMANOVA, Jaccard distance: *R*^*2*^ = 0.119, *F* = 4.000, *p* = 0.001; beta dispersion *F* = 38.093, *p* = 0.001).

Overall, wild mice had a higher ratio of Firmicutes to Bacteroidota than lab mice (Fig. 2B). At the bacterial family level, the lab mouse microbiota was dominated by Muribaculaceae (mean relative abundance 46.7%, standard deviation 14.1%), while wild mice did not show consistent dominance by a single family but typically had higher relative abundance of Lactobacillaceae and Lachnospiraceae (Fig. 2C). In a Random Forest regression model, the ten ASVs most important for distinguishing wild from lab mice all had either very low or zero relative abundance in wild mice and belonged to the bacterial families Akkermansiaceae, Muribaculaceae, and Desulfovibrionaceae (Out-of-bag (OOB) estimate of error = 0%; Fig. S5 A). These taxa enriched in lab mice did not appear to be related to a specific strain or animal facility (Fig. S5 B).

### Gut microbiota turnover rate is faster in wild than laboratory mice

**Fig. 3:**
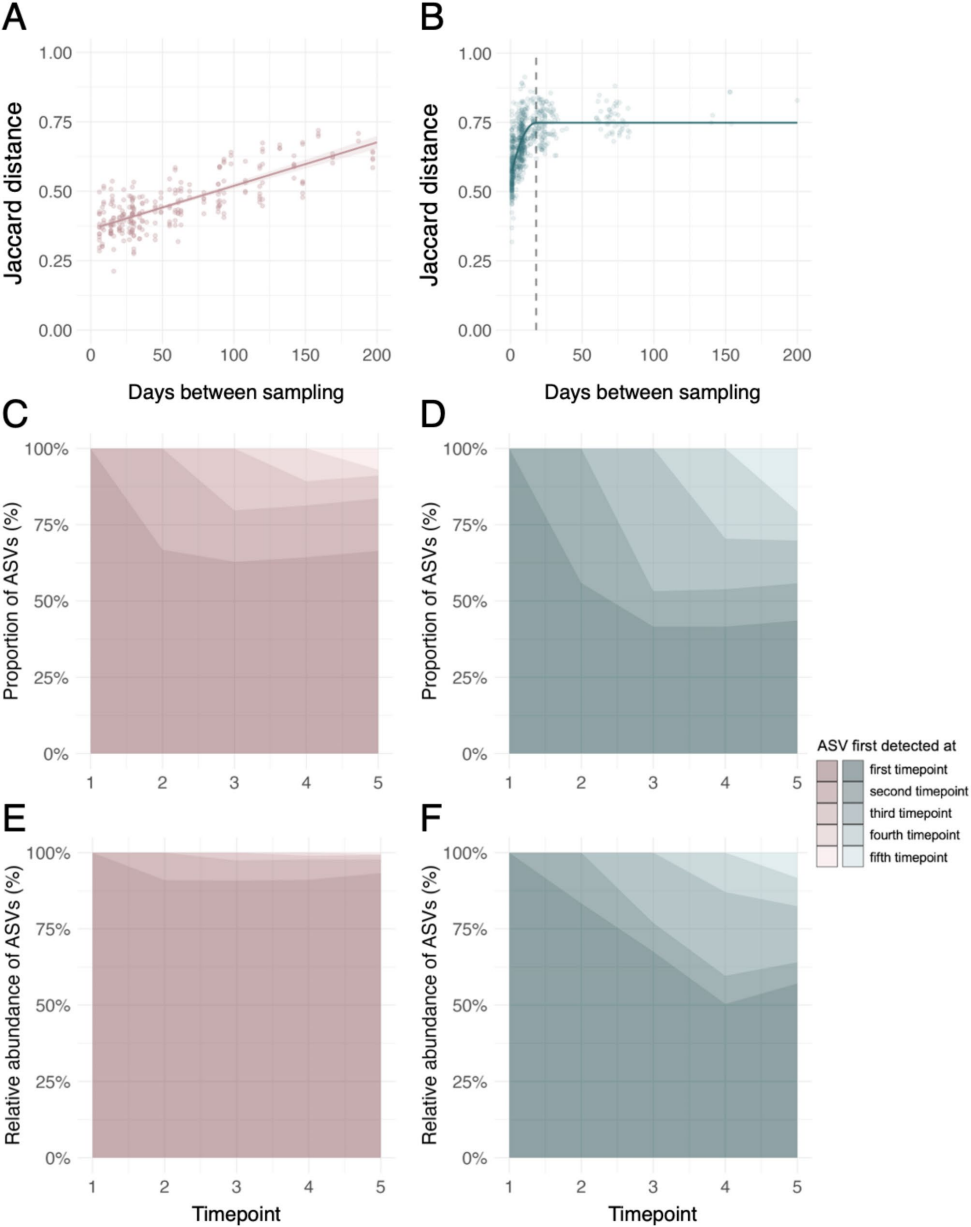
Gut microbiota turnover in laboratory (*left*) and wild (*right*) mice. (**A**–**B**) Within-individual microbiota distance on Jaccard distance between sample pairs from the same (**A**) laboratory or (**B**) wild mouse. (**A**) Lab mice: Linear model; *R*^*2*^ *=* 0.605, *F*_1,254_=391.5, *p* < 0.001; all samples from repeat-sampled laboratory mice from C57BL/6 colony from Animal Facility B, *n* = 99; 5–7 samples from 16 animals. (**B**) Wild mice: quadratic plateau model; *R*^*2*^ *=* 0.494, critical point of inflexion = 17.9 days; all samples from repeat-sampled Skokholm Island mice without exclusion based on season or age, *n* = 555; 2–10 samples from 179 animals. (**C**–**D**) Mean proportion and (**E–F**) mean relative abundance of amplicon sequence variants (ASVs) at five timepoints in (**C**, **E**) laboratory (*n* = 5) and (**D**, **F**) wild mice (*n* = 5) based on the timepoint the ASV was first detected. Laboratory and wild mice for which similar sampling intervals (5 samples ~ 1 week apart) were available were selected for the analysis. Timepoints are days 0, 9, 15, 23, and 29 for laboratory mice and days 0, 6–8, 12–16, 22–24, and 30–32 for wild mice

We hypothesised that wild mice, exposed to a more diverse and variable microbial pool than lab mice, would experience higher within-host turnover in gut microbial taxa. Consistent with this hypothesis, in lab mice, gut microbial turnover increased gradually up to a sampling interval of 200 days without reaching a plateau (linear model; *R*^*2*^ *=* 0.605, *F*_1,254_=391.5, *p* < 0.001; Fig. 3A), while in wild mice, microbial turnover increased among sample pairs less than 20 days apart, but then reached a plateau (quadratic plateau model; *R*^*2*^ *=* 0.494, critical point of inflexion = 17.9 days; $\:\varDelta\:$AIC vs. linear model: 334.1; Fig. 3B). While both lab and wild mice appeared to possess a similar maximal level of turnover of 75% distinct ASVs between timepoints, our findings suggest wild mice reach this level much faster. Additionally, in lab mice, gut microbial turnover seemed to be related to cage density, with mice housed at higher density exhibiting a slower rate of microbiota turnover over time (Fig. S6). This suggests that cage-associated microbial transmission may play a role in stabilising the gut microbiota in laboratory conditions, potentially buffering against external microbial influences.

The observed faster microbial turnover in wild mice can also be observed by considering ASV changes during a fixed one-month period in both systems. In a set of five repeat-sampled lab mice, ~ 70% ASVs detected at the start of the month were still detected at the end. By contrast, among five wild mice also sampled over a month, this was true of only 45% ASVs (Fig. 3C–D). Similarly, around 95% of total microbial relative abundance in the lab mouse microbiota present at the end of one month comprised ASVs present at the start, whereas for wild mice this was only 60% (Fig. 3E–F). While these patterns varied slightly across individuals, retention of ASVs over time was consistently higher in lab mice (Fig. S7). Among these lab mice, there were 35 ‘persistent’ ASVs (those detected at all timepoints in all five mice) that originated from ten bacterial families (Muribaculaceae, Rikenellaceae, Marinifilaceae, Oscillospiraceae, Bacteroidaceae, Akkermansiaceae, Lachnospiraceae, Sutterellaceae, Tannerellaceae, and Erysipelotrichaceae). Their combined relative abundance was 41–79% (mean 62%) in these longitudinally studied mice. These 35 ASVs were relatively common in the wider colony, with all 35 detected in 26% samples (32 of 123) and ≥ 33 detected in 83% samples (102 of 123). In contrast, only 8 persistent ASVs were identified in the five wild mice, belonging to the families Lactobacillaceae (*n* = 1), Oscillospiraceae (*n* = 5) and Lachnospiraceae (*n* = 1), and Muribaculaceae (*n =* 1). These persistent ASVs had a combined relative abundance of 3–23% (mean 9%) in these five mice, and were less common in the wider population, with all 8 detected in 50% samples (412 of 903) from the Skokholm population as a whole. Overall, these results suggest that the gut microbiota changes faster in the wild than in the lab, with wild mice harbouring fewer temporally persistent taxa that together make up a lower cumulative relative abundance than temporally persistent taxa in lab mice.

### Aerotolerant gut bacteria are more abundant and richer in wild mice than lab mice

**Fig. 4:**
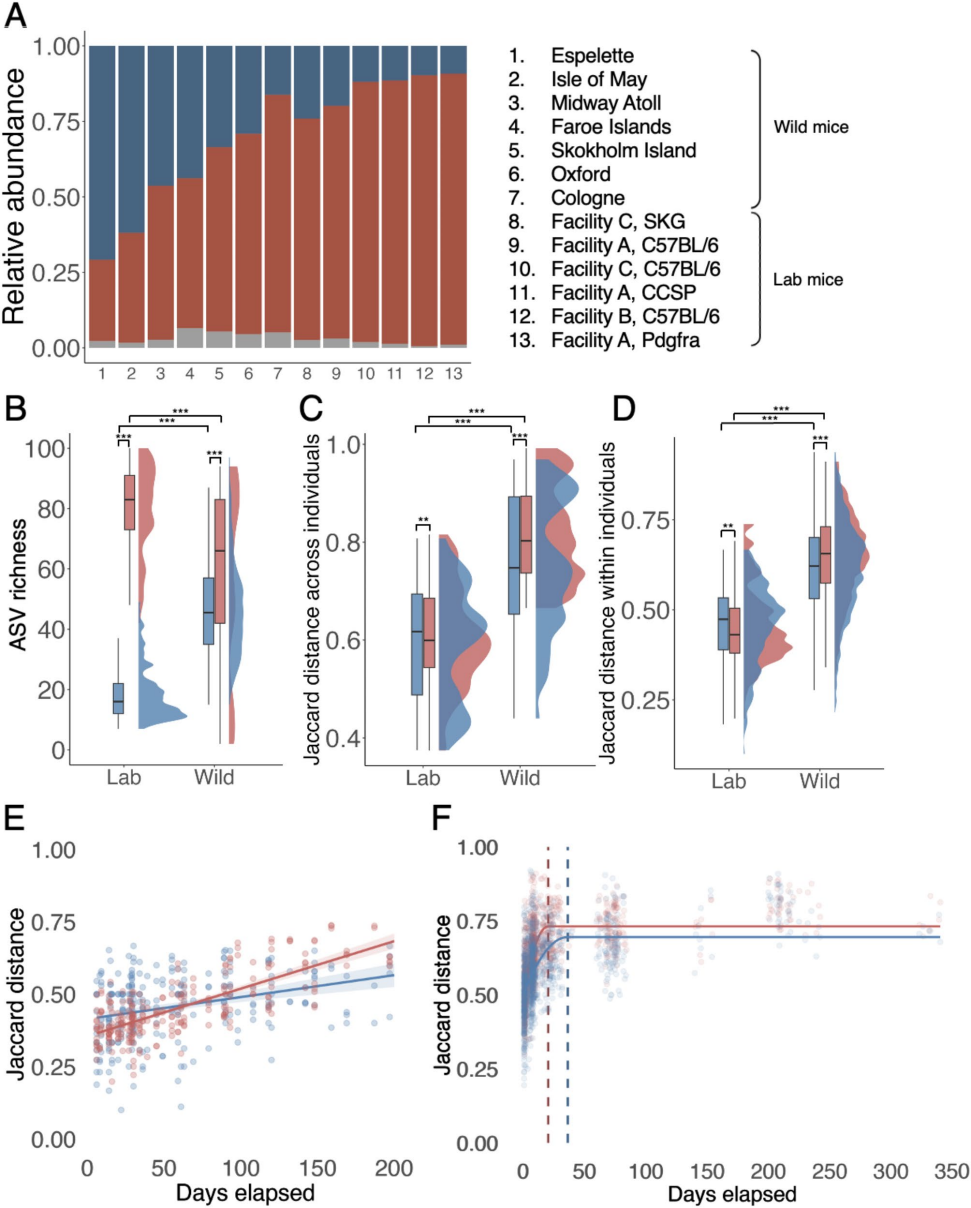
Patterns of aerotolerance in lab and wild mouse gut microbiota. Colour indicates bacterial aerotolerance: *red* = obligate anaerobes, *blue* = all other bacteria with known aerotolerance (aerotolerants), *grey* = bacteria with unknown aerotolerance. (**A**) Mean relative abundance of aerotolerant and anaerobic bacteria across six wild populations (180 samples in total) and five laboratory colonies (146 samples in total). Bars are individual populations/colonies. (**B**) ASV richness within anaerobic and aerobic communities in lab (*n* = 146) and wild (*n* = 180) mice. Statistical differences were tested with Wilcoxon rank sum tests (***; *p* < 0.001). (**C**) Pairwise Jaccard distance across individuals from a given lab colony or wild population. Statistical differences were tested with permutational Wilcoxon rank sum tests (***; *p* < 0.001). (**D**) Pairwise Jaccard distance within individuals sampled over time in lab (Facility B, C57BL/6 strain; 99 samples from 16 individuals, 5–7 samples per individual) or wild (Skokholm Island; 717 samples from 217 individuals, 2–12 samples per individual). Statistical differences were tested with permutational Wilcoxon rank sum tests (***; *p* < 0.001). Equal sample sizes were achieved by randomly sampling based on the lower number of pairwise data points (*n* = 1024 random pairwise data points from each system). (**E**–**F**) Within-individual microbiota dissimilarity on Jaccard distance between sample pairs from the same mouse for (**E**) laboratory mice (99 samples from 16 individuals, 5–7 samples per mouse) and (**F**) wild mice (717 samples from 217 individuals, 2–12 samples per individual) against time between samples. Laboratory mice were from a single C57BL/6 colony (Animal facility B) and wild mice from Skokholm Island (Table S1). The relationship is fitted with (**E**) a linear model (lab mice: *anaerobic taxa*: *R*^*2*^ *=* 0.520, *F*_1,254_=277.2, *p* < 0.001; *n* = 99; 5–7 samples from 16 animals; *aerotolerant taxa: R*^*2*^ = 0.101, *F*_1,254_=29.78, *p* < 0.001) and (**F**) a quadratic plateau model (wild mice: *anaerobic taxa*; adjusted *R*^*2*^ = 0.493, critical point of inflexion = 20 days, $\:\varDelta\:$AIC vs. linear model: 499.0; *aerotolerant taxa*; adjusted *R*^*2*^ = 0.311, critical point of inflexion = 37 days, $\:\varDelta\:$AIC vs. linear model: 148.7). Critical points of inflection are indicated by dashed vertical lines

We next investigated whether gut microbial aerotolerance patterns vary between wild and lab mice. On average, wild mice had a significantly higher proportion of aerobes out of taxa with known aerotolerance than lab mice with only one wild population (Cologne) having a lower ratio than a lab colony (Fig. 4A; brm model: posterior mean for *wild*: 1.75, CIs 0.79 to 2.72). In lab mice, aerotolerant bacteria made up around 11% of the gut microbiota (mean relative abundance 11.2%, median 9.7%, range 2.5–35.8% across samples), whereas this was on average around 3 times higher among wild mice (mean 37.7%, median 32.2%, range 7.1–99.9%; aerobes formed > 90% relative abundance in a total of five samples from Espelette and Faroe populations). Despite the higher proportion of Proteobacteria in wild mice, including taxa from the Gammaproteobacteria class, the observed difference in aerotolerant taxa between wild and lab mice is not solely driven by this group. When Gammaproteobacteria were excluded, the proportion of aerotolerant taxa remained nearly unchanged (11% in lab mice vs. 36% in wild mice), indicating that other factors contribute to this pattern.

In both wild and lab mice, anaerobic taxa showed higher diversity than aerobic taxa (Fig. 4B). Wild mice also harboured a higher diversity of aerotolerant taxa than lab mice, but the opposite pattern was observed for obligate anaerobic taxa, which were more diverse in lab mice (Fig. 4B). Beta diversity of both anaerobic and aerobic communities was consistently higher in wild compared to lab mice both across and within individuals (Fig. 4C, D). In wild mice, the anaerobic community had slightly greater variability in composition (beta diversity) among individuals as well as within individuals, whereas in lab mice the opposite pattern was observed (Fig. 4C, D). Within repeat-sampled wild and lab mice, anaerobic bacteria exhibited a higher rate of turnover over longer time periods (Fig. 4E and F). However, in lab mice, aerotolerant communities showed greater variation when considering all timepoints together (Fig. 4D), suggesting that in the lab their composition fluctuates more over short time windows, whereas anaerobic taxa shift more gradually but persistently over time.

One methodological factor that might increase the richness and abundance of aerotolerant taxa in wild compared to lab mice is an increased exposure to outdoor, high oxygen conditions prior to sample collection (if samples are collected from traps left overnight). We assessed the potential influence of this methodological factor by modelling whether the duration of time before a wild mouse faecal sample was collected and preserved in DNA/RNA Shield predicted either the richness of aerotolerant taxa or the proportion of aerobes out of taxa with known aerotolerance among mice for which this data was available (all from Skokholm population). We found no evidence that the duration before sample collection influenced either the proportion of aerobes out of all taxa with known aerotolerance (brm model: posterior mean 0.95, CIs -1.19 to 3.12; *n* = 48) or the ASV richness of aerotolerant (posterior mean: 1.49, CIs -0.42 to 3.30) or anaerobic taxa (posterior mean -0.53, CIs -2.76 to 1.76).

### Island mice have a microbiota that differs from mainland mice and diverges more from the lab mouse microbiota

**Fig. 5:**
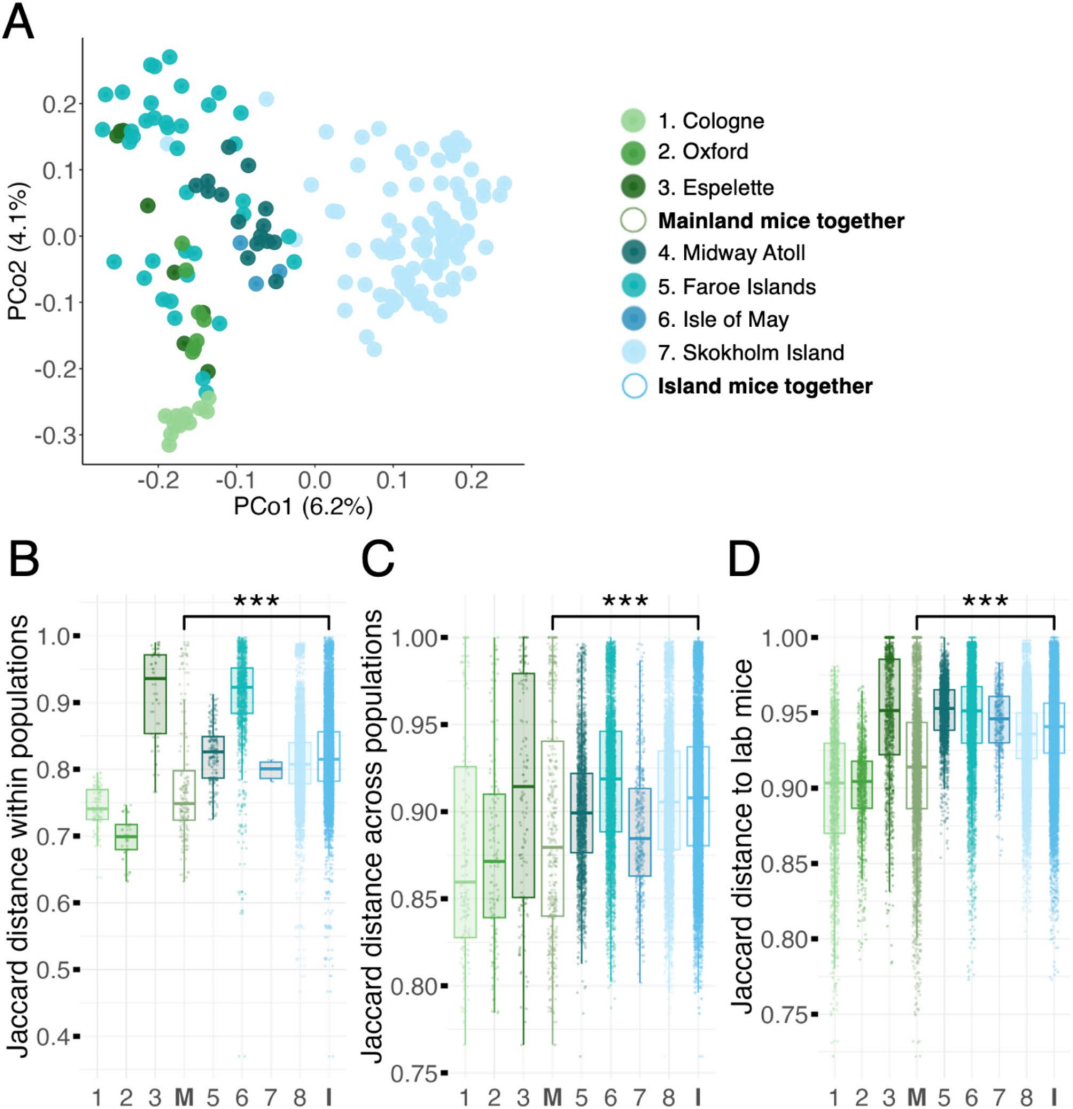
(**A**) Principal coordinates analysis of Jaccard distance among mouse gut microbiotas from three mainland (Cologne, *n* = 11; Oxford, *n* = 7; Espelette, *n* = 8) and four island populations (Midway Atoll, *n* = 15; Faroe Islands, *n* = 38; Isle of May, *n* = 3; Skokholm, *n* = 98). (**B**) Pairwise Jaccard distance between samples from the same mainland or island population. (**C**) Pairwise Jaccard distance between samples from different mainland or island populations. Each population-specific box depicts dissimilarity values from members of that population to members of all other populations of the same type (island or mainland). (**D**) Pairwise Jaccard distance between samples from wild populations and samples from lab mice (3–98 samples per wild mouse population, 146 samples from lab mice)

We next investigated variation in the gut microbiota among wild mouse populations, specifically between mainland and island populations. Wild mice from mainland vs. island populations showed some distinction in the composition of their gut microbiota, but this was relatively subtle, with setting (mainland vs. island) explaining ~ 3% variance in composition regardless of whether bacterial phylogeny was considered or not (PERMANOVA, Jaccard distance: *R*^*2*^ = 0.026, *F* = 4.846, *p* = 0.001; beta dispersion *F* = 2.321, *p* = 0.146; UniFrac distance, *R*^*2*^ = 0.030, *F* = 5.806, *p* = 0.001; beta dispersion *F* = 0.053, *p* = 0.824). In principal coordinates analyses (PCoA), there was no clear clustering by setting irrespective of the distance metric used (Jaccard, Aitchison, unweighted and weighted UniFrac; Fig. 5A, Fig. S8). A Random Forest regression model was used to identify which ASVs distinguish mainland and island mice. The top ten ASVs all had a higher relative abundance in mainland than island mice and belonged to the orders Bacteroidales, Lachnospirales, Oscillospirales, and Lactobacillales (OOB estimate of error = 3.9; Fig. S9). The second most important taxon was *Muribaculum intestinale*, which was also an important driver of the lab/wild distinction, with higher relative abundance in lab than wild mice (Fig. S5).

Overall, the gut microbiota of island-dwelling mice was more variable within populations than mainland mice (Fig. 5B), and island populations also had microbiotas that were more distinct from one another than mainland populations (Fig. 5C). These patterns were similar for subsets of aerotolerant and anaerobic taxa (Fig. S10). Finally, mainland mice had a gut microbiota composition that was more similar to lab mice than island-dwelling mice, though Espelette was somewhat of an outlier, with mice from this mainland population sharing fewer taxa with lab mice than other mainland populations (Fig. 5D).

## Discussion

Using over 850 samples from 346 individual mice across multiple laboratory facilities and strains as well as both mainland and island natural populations, we demonstrate that lab and wild mice harbour taxonomically and functionally distinct gut microbiotas that vary in their average aerotolerance and show different temporal dynamics. We further show that mice on oceanic islands harbour gut microbiotas that are distinct from those of mainland mice, as well as more distinct from lab mice.

In line with previous studies [10, 13, 46], we find that the gut microbiota of wild mice was on average more taxonomically diverse than that of lab mice, although alpha diversity varied greatly between laboratory colonies and wild mouse populations. This included variation between laboratory strains from the same animal facility and within a single strain (C57BL/6) from different facilities, similar to previous findings [11], further highlighting the importance of controlling for such covariates in lab studies to ensure experimental reproducibility [47]. Most of the heightened gut microbiota richness in wild mice derives from aerotolerant taxa. This may reflect their natural exposure to diverse environmental microbes, in contrast to lab mice, whose sterile living conditions (e.g., routine sterilisation of food and cages) limit contact with aerotolerant taxa.

The number of unique functional pathways was also higher in the microbiota of wild compared to lab mice. This increased diversity could arise from wild mice having a more diverse diet than lab mice (favouring microbial taxa with a broader functional capacity than those favoured by homogenous laboratory diets) [48], or from exposure of wild mice and their microbes to a wider range of environmental conditions (e.g., temperature, water availability, or salinity) compared to lab mice housed under highly stable conditions [49, 50].

As is generally observed in mammals, the predominant phyla in both wild and laboratory mice were Firmicutes and Bacteroidota. Here we found that wild mice tended to have a higher ratio of Firmicutes to Bacteroidota, contrasting with the findings of Rosshart et al. (2017) [12] where the opposite pattern was observed. We also found a higher relative abundance of Proteobacteria in wild mice compared to lab mice (as previously identified [12]), which may be due to the exclusion of common pathogens (e.g., *Salmonella*) from specific-pathogen-free (SPF) laboratory facilities.

Contrary to observations in Thomson et al. (2022) [18], we detected Akkermansiaceae, Streptococcaceae, and Enterobacteriaceae not only in lab but also in wild mice. In particular, Streptococcaceae and Enterobacteriaceae occurred at high prevalence across wild mouse samples (~ 80% and ~ 55%, respectively). These families had low relative abundance in most wild populations (0.002–3.0%) but were more common in others (~ 17% and ~ 11% in Espelette and Midway Atoll populations, respectively). Muribaculaceae, which is considered an important family in the mouse gut [51–53], dominated lab mice (~ 47%) but was less abundant in wild mice (~ 14%). Consistent with this, Muribaculaceae taxa were key in driving microbiota differences between lab and wild mice, in line with Bowerman et al. (2021) [11]. One such taxon was *Muribaculum intestinale*, which was omnipresent in lab mice but rare in wild mice (detected in 14% of wild mouse samples, with < 1% mean relative abundance). These findings underscore the need for cross-populational studies to capture the full ecological diversity of the house mouse gut microbiota.

Overall, the gut microbiota was more variable among individuals of wild than lab mice, and the within-host turnover of gut microbial taxa was faster in wild mice. In lab mice, microbiota turnover increased gradually over 200 days without reaching a plateau, whereas in wild mice, turnover plateaued after just 20 days. We also found that microbiota turnover was slower in lab mice housed at higher cage density, suggesting that microbial transmission between cage mates contributes to microbiota stability. This result was based on a limited sample, as all high-density mice were housed in a single cage, making it difficult to separate density effects from cage-specific influences. These patterns are perhaps expected given the broader and more variable microbial transmission sources and environmental conditions experienced in the wild. We further found that taxa persisting within individuals over time were also prevalent across individuals, with this core of ‘persistent’ microbes being larger in lab than wild mice, reflecting the more dynamic microbiota of wild mice.

Wild and lab mice also differed in the dynamics of aerotolerance in their microbiotas. Overall, wild mice harboured a higher relative abundance of aerotolerant bacteria, but there was substantial variation across wild populations. Within wild mice, anaerobic bacteria were more variable across individuals than aerotolerant bacteria, whereas in lab mice, the opposite pattern was observed. Over time, anaerobic bacteria changed more in wild mice regardless of sampling window, whereas in lab mice, the anaerobic community showed higher turnover over longer time periods, while the aerotolerant community fluctuated more over shorter time windows. These differences between lab and wild mice may reflect differences in microbial transmission routes. Anaerobic bacteria, which cannot persist long outside the host, likely require direct transmission via maternal or social contact. Their distribution may therefore follow host social networks rather than spatial proximity. In wild mice, spatial connectivity may drive homogenisation of aerotolerant bacteria across the population, while social transmission leads to more distinct anaerobic communities [54]. In lab mice, anaerobes are primarily acquired at birth and transmitted among a small, stable set of cage mates, which may initially lead to a homogenised community. However, over longer time periods, the anaerobic microbiota gradually shifts, possibly due to host-level changes or sporadic introduction of new strains from handlers. In contrast, aerotolerant bacteria may be introduced more randomly through occasional contamination from the relatively sterile lab environment, leading to frequent but transient fluctuations in composition over shorter time windows.

Together these findings indicate that, in comparison to wild mice, the lab mouse gut microbiota has a slow turnover rate and differs in its aerotolerance patterns likely due to variation in transmission processes. Understanding transmission dynamics and temporal patterns in the lab mouse gut microbiota may be particularly important when developing therapeutics aimed at altering the gut microbiota as a community. Due to their slowly but gradually shifting microbiota that has a relatively low diversity, therapeutics such as faecal microbiota transplants could elicit more pronounced and controllable effects in lab mouse recipients, but weaker and perhaps more unpredictable alterations in wild mouse recipients given their more natural microbiota, that is more diverse and dynamic and thus more resilient to change.

While assigning aerotolerance based on 16 S taxonomy provides useful ecological insights, it is inherently limited by the resolution of amplicon sequencing (typically genus-level), while oxygen tolerance can vary among species within a genus. This lack of resolution has potential to introduce bias to the patterns observed. Future work incorporating metagenomics or functional profiling could help refine these ecological inferences.

Finally, we explored gut microbiota variation among wild mouse populations. House mice are one of the most successful invasive species and have colonised many islands [55–58] where they are often of larger body size [59]. Wild mouse microbiotas showed some signature of the population from which they originated, though this was not strong. Even mice from Midway Atoll in the North Pacific Ocean, introduced over 75 years ago [60] and located more than 10,000 km from other sampled populations, did not exhibit a clearly distinct gut microbiota from other wild mice. However, gut microbiota composition did vary somewhat between island and mainland populations, with certain taxa distinguishing mainland mice from island mice. This could be due to convergent environmental factors and therefore selection pressures on microbes colonising mice on oceanic islands, such as similar diets [61] or similar host selection for increased body size, as commonly observed among island mice [62]. Another potential factor could be variation in the extent of anthropogenic influence, which may be why mice on oceanic islands had a microbiota composition more divergent from that of laboratory mice than that of mainland mice. We also observed that the gut microbiota of island-dwelling populations differed more in composition from one another than mainland populations, consistent with the higher isolation of island mouse populations providing greater potential for their microbiota to diverge. Future studies that investigate microbiota variation in species like house mice that have invaded multiple islands and diversified on them would be valuable, to understand the patterns of microbiota divergence and what role different microevolutionary processes (e.g., drift, divergent ecological selection, and host–microbe co-diversification) play in this.

Overall, we demonstrate that wild house mice harbour a gut microbiota that is taxonomically and compositionally distinct from that of laboratory mice, and that displays higher aerotolerance and much faster taxonomic turnover. We also provide early insights into gut microbiota variation across wild mice in different geographic settings, that suggest subtle differences in gut microbiota composition and variability between mainland and island-dwelling mice.

## Electronic supplementary material

Below is the link to the electronic supplementary material.

Supplementary Material 1

## Data Availability

The 16 S rRNA amplicon sequencing data used in this study have been deposited in GenBank under SRA accession: PRJNA1187891. Sample specific metadata, aerotolerance data and R code used in the study is publicly available at https://github.com/eveliinahanski/mus_microbiota.

## Abbreviations

PIT: Passive integrated transponder
PERMANOVA: Permutational multivariate analyses of variance
CLR: Centred log ratio
ICAC: Islands conservation advisory committee
USFWS: U.S. fish and wildlife service
OOB: Out of-bag
SPF: Specific pathogen-free
