# Supplementary material for: Wild house mice have a more dynamic and aerotolerant gut microbiota than laboratory mice
Source: BMC Microbiol. 2025 Apr 9;25:204. doi: 10.1186/s12866-025-03937-1 (PMC11983872; doi:10.1186/s12866-025-03937-1)
Supplement: Supplementary file 1 — Supplementary Material 1 [file 12866_2025_3937_MOESM1_ESM.docx]

**Supplementary data**

**Supplementary Table 1.** Origin, sample type and preservation of samples used in the study. Samples were shipped on dry ice unless preservative allowed sample shipping at room temperature (DNA/RNA Shield). BMS, Biomedical Services Building.

| **Source** | **Sub-species, strain (*abbreviation*)** | **Intervention** | **Facility/population** | **Sampling dates** | **Sample type** | **Preservative, storage temperature** | **Sample size for cross-sectional (*longitudinal*) analyses** |
| --- | --- | --- | --- | --- | --- | --- | --- |
| Laboratory | C57BL/6 | No | Animal facility A (BMS, Oxford, UK) | Oct 2021 | Faeces | DNA/RNA Shield, -80^o^C | 15 |
| Laboratory | C57BL/6 | No | Animal facility B (King’s College, London, UK) | May–Nov 2021 | Faeces | DNA/RNA Shield, -80^o^C | 112, (*99*) |
| Laboratory | C57BL/6 | No | Animal facility C (Kennedy Institute, Oxford, UK) | Nov 2020 | Faeces | DNA/RNA Shield, -80^o^C | 6 |
| Laboratory | SKG | Curdlan induced intestinal inflammation | Animal facility C (Kennedy Institute, Oxford, UK) | Nov 2020 | Faeces | DNA/RNA Shield, -80^o^C | 3 |
| Laboratory | Pdgfra-CreER (*Pdgfra*) | No | Animal facility A (BMS, Oxford, UK) | Dec 2020 | Faeces | DNA/RNA Shield, -80^o^C | 6 |
| Laboratory | CCSP-rtTA (*CCSP)* | No | Animal facility A (BMS, Oxford, UK) | Dec 2020 | Faeces | DNA/RNA Shield, -80^o^C | 4 |
| Wild | *Mus musculus domesticus,* N/A | No | John Krebs Field Station, Wytham, UK | Nov 2020 | Faeces | DNA/RNA Shield, -80^o^C | 7 |
| Wild | *Mus musculus domesticus,* N/A | No | Skokholm Island, Wales, UK | Apr–May 2019; July 2019; Sept–Oct 2019; Aug–Sept 2020; Apr–May 2021 | Faeces | DNA/RNA Shield, -20^o^C/-80^o^C | 98, (*555*) |
| Wild | *Mus musculus domesticus*, N/A | No | Isle of May, Scotland, UK | Aug–Oct 2018 | Faeces | None, -80^o^C | 3 |
| Wild | *Mus musculus domesticus*, N/A | No | Cologne, Germany | Aug–Sept 2009 | Intestinal contents (cecum) | RNAlater, -80^o^C | 11 |
| Wild | *Mus musculus domesticus*, N/A | No | Espelette, France | Aug/Sept 2013 | Intestinal contents (colon) | PBS, -80^o^C | 8 |
| Wild | Admixture of *Mus musculus* subspecies (*domesticus*, *musculus*, and *castaneus*), but mostly *M. m. domesticus*^61^, N/A | No | Midway Atoll, North Pacific Ocean | Oct–Nov 2018 | Intestinal contents (colon) | 70% isopropyl alcohol until moved to DNA/RNA Shield for shipping from US to the UK, -20^o^C/-80^o^C | 15 |
| Wild | *Mus musculus* ssp., mainly *M. m. domesticus*^62^*,* N/A | No | Faroe Islands, North Atlantic Ocean |  | Intestinal contents (colon) | None until moved to DNA/RNA Shield for shipping from Faroe Islands to the UK, -20^o^C | 38 |


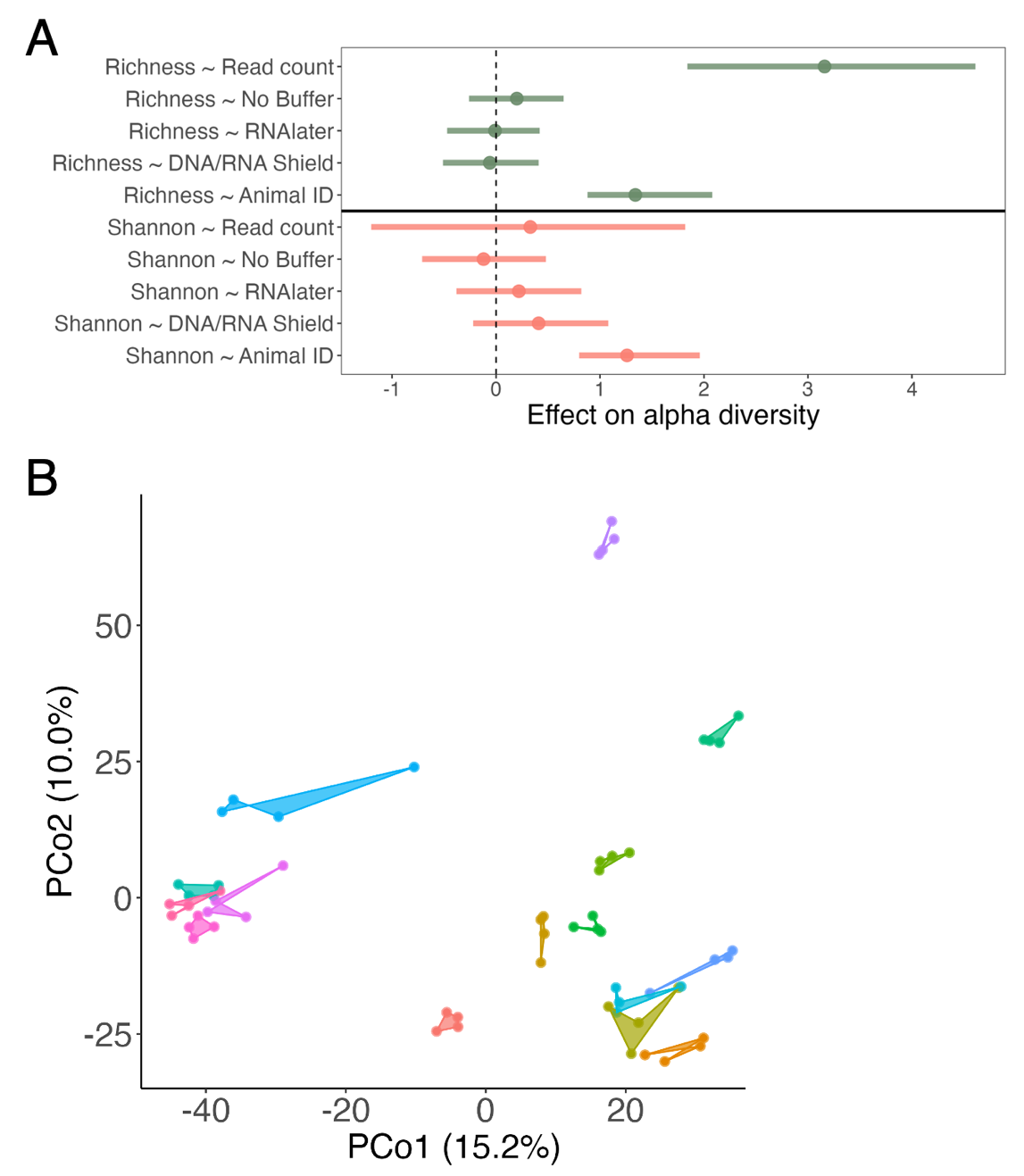


**Supplementary Figure 1.** Estimated effect of sample preservative on gut microbiota (**A**) diversity and (**B**) composition in 15 wild mouse faecal samples sampled on Skokholm Island. Replicate aliquots were stored in either DNA/RNA Shield, RNAlater, absolute ethanol, or without preservative. (**A**) Estimated effects on alpha diversity from Bayesian regression (brm) models, with predictors including storage system (reference: absolute ethanol), read count, and animal ID. Green represents asymptotic ASV richness, and red represents asymptotic Shannon diversity. Alpha diversity estimates and read count were scaled to 0–1 for interpretability. Points indicate posterior means and lines show 95% credible intervals (CIs). A variable significantly predicts alpha diversity if its credible interval does not overlap zero, and effects are significantly different if their credible intervals do not overlap. ASV richness and Shannon diversity were modelled separately, meaning estimates between these two are not directly comparable. (**B**) Principal coordinate analysis of four aliquots from 15 Skokholm mice on Aitchison distances. Aliquots were stored in DNA/RNA Shield, RNAlater, absolute ethanol or without preservation buffer. Colours represent individual mice, points are different aliquots.

**
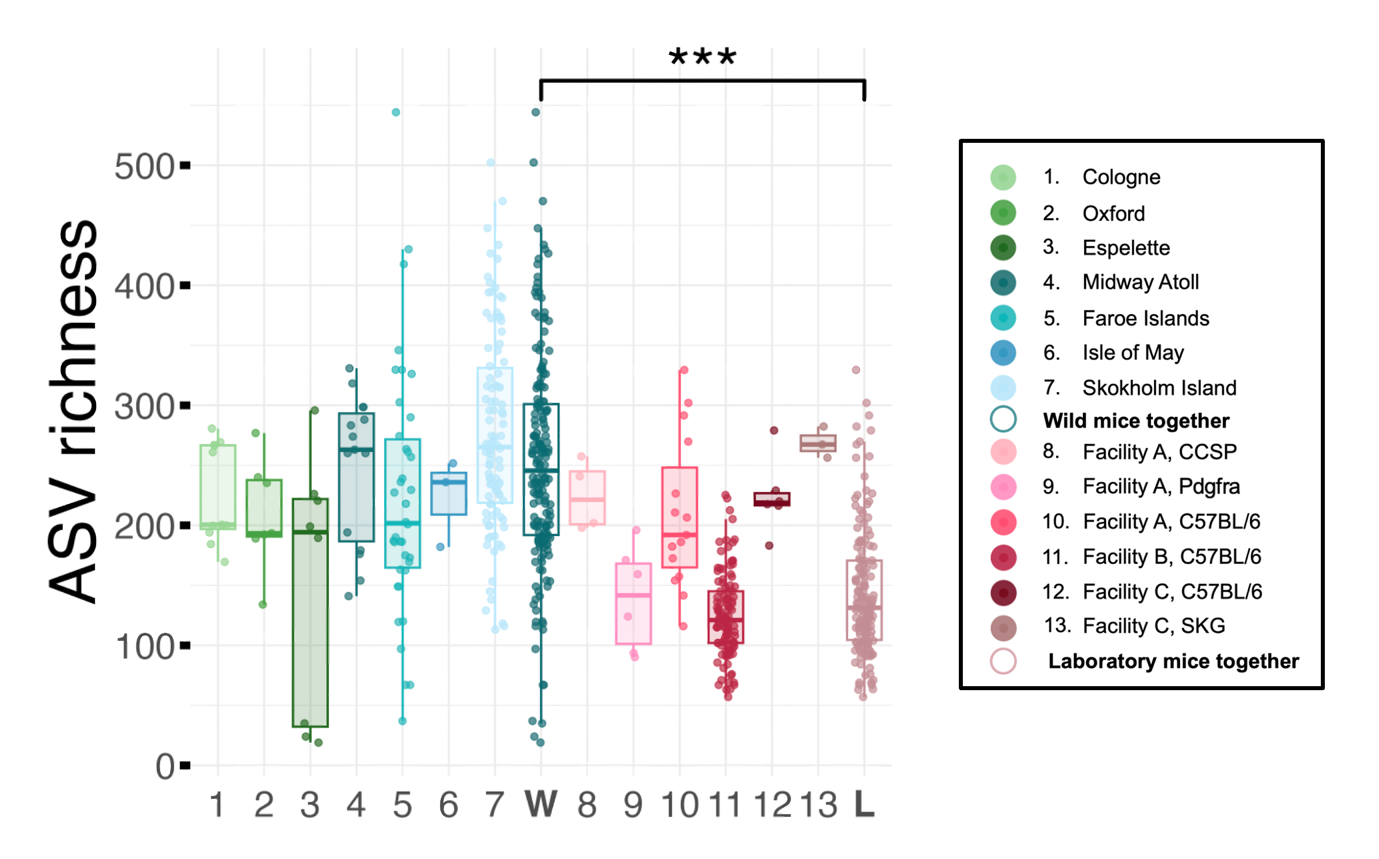
Supplementary Figure 2.** Asymptotic ASV richness in wild (*n*=436) and laboratory (*n*=146) mice. Boxplots are individual wild mouse populations (mainland populations, *green*; island populations, *blue*) or laboratory mouse colonies (*pink*). Empty boxes represent all wild (*teal,* ‘W’) or laboratory (*pink,* ‘L’) mouse samples pooled. Statistical differences between lab and wild mice were tested with permutational Wilcoxon rank sum tests (***; *p*<0.001).


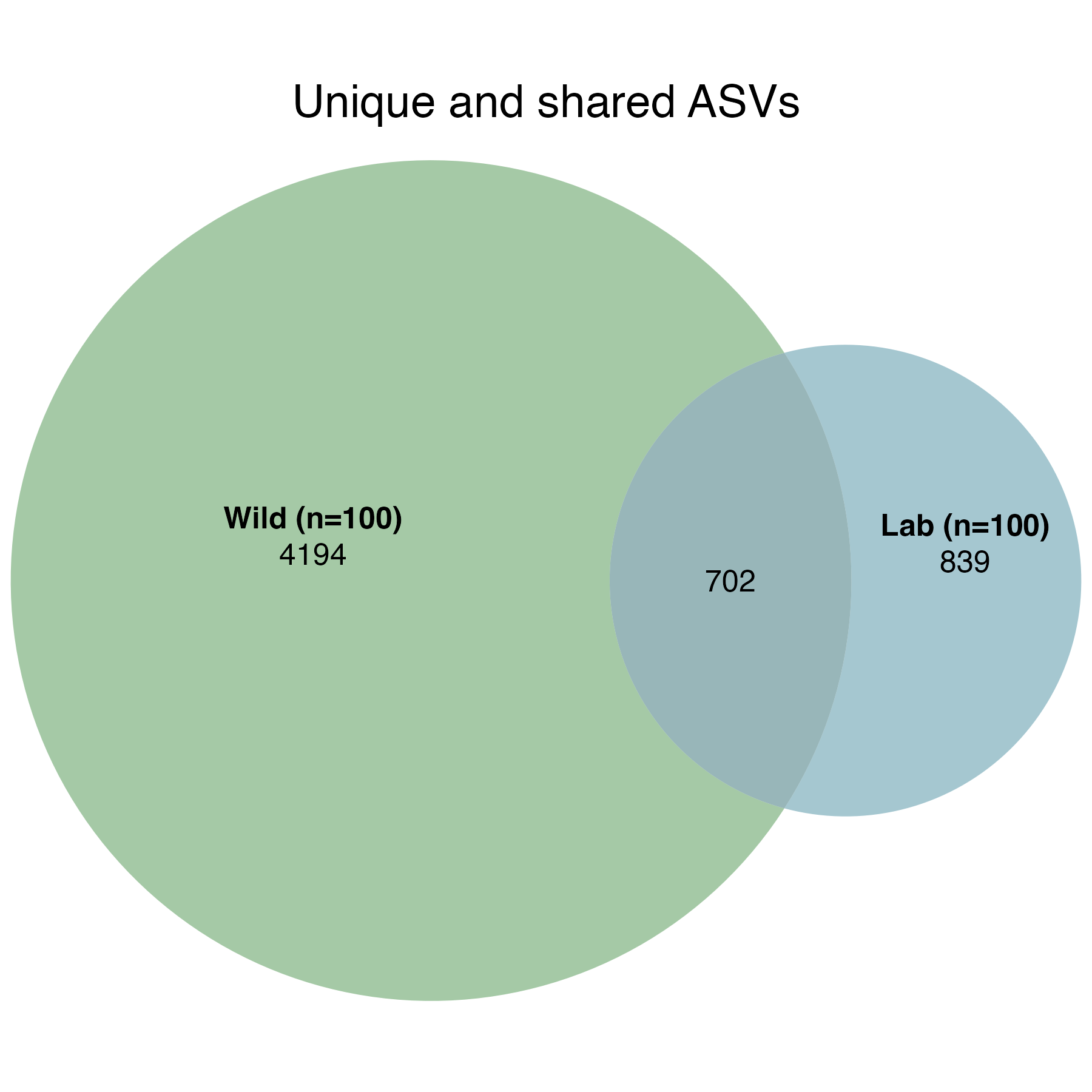
**Supplementary Figure 3.** Unique and common ASVs in a random subset of 100 wild and 100 lab mouse samples (randomly selected from a total of 180 wild and 140 lab mouse samples).


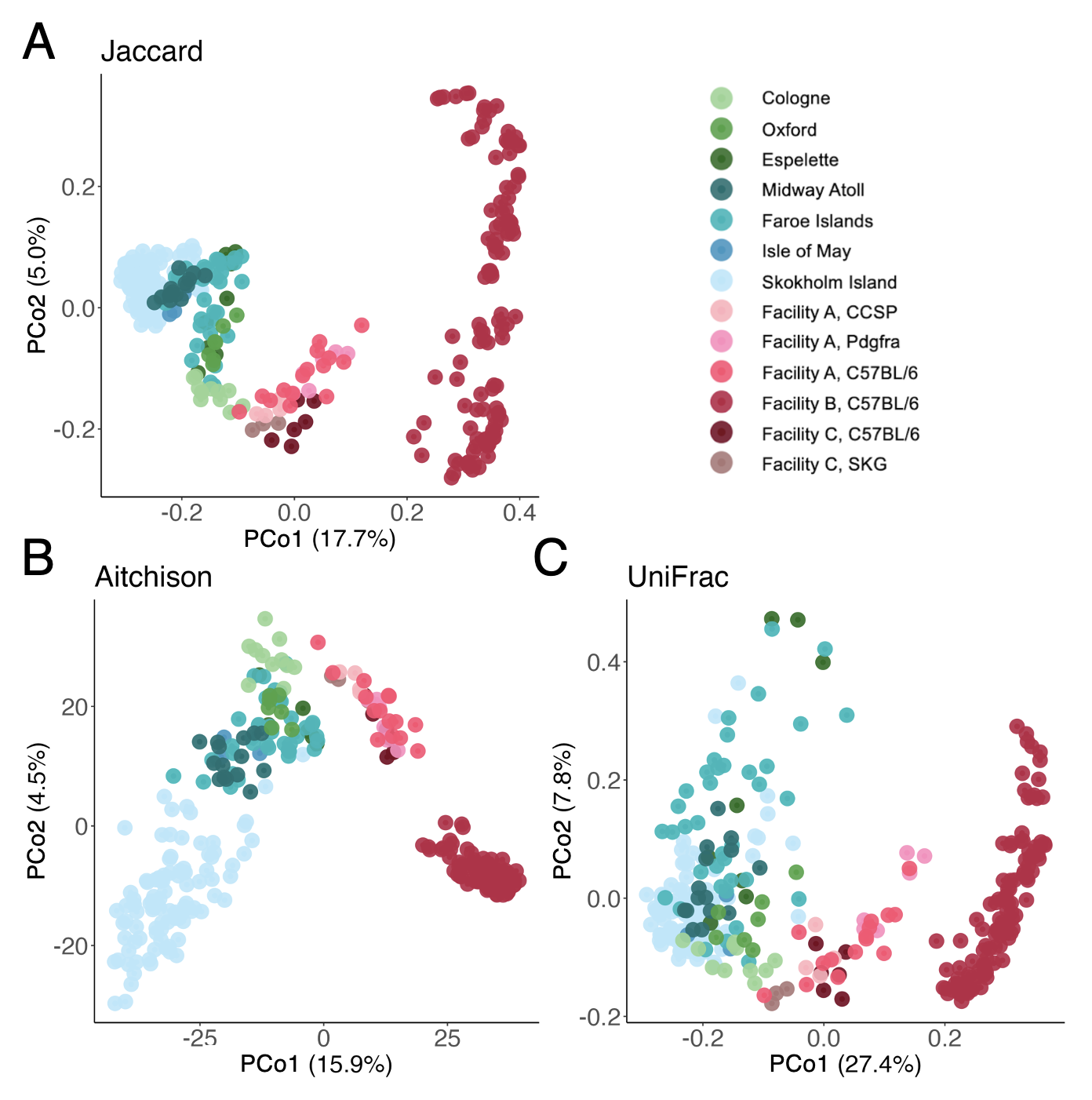


**Supplementary Figure 4.** (**A–C**) Principal coordinate analysis (PCoA) of wild and laboratory samples on (**A**) Jaccard, (**B**) Aitchison, and (**C**) unweighted UniFrac distances from seven wild populations and six laboratory colonies.


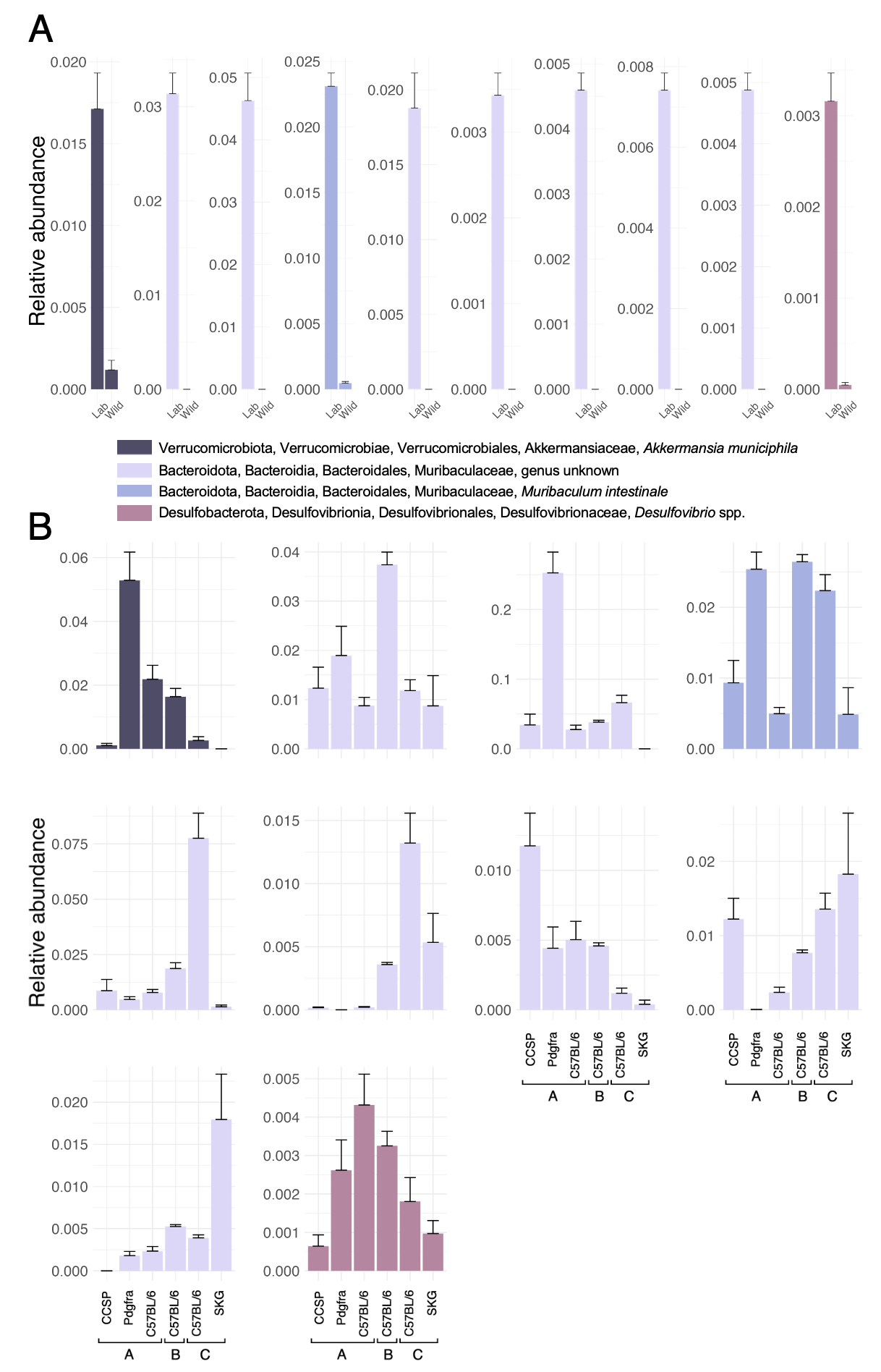


**Supplementary Figure 5.** (**A**) Relative abundance of top ten amplicon sequence variants (ASVs) predicting source (lab/wild), ordered in decreasing order of importance from left to right. ASVs were identified with Random Forest regressions, with mean decrease in Gini used as a measure of importance, such that the higher the score, the higher the importance (OOB estimate of error = 0%). (**B**) Relative abundance of the top ten ASVs across four lab strains from three animal facilities. Letters A, B, and C on x-axis refer to animal facilities.


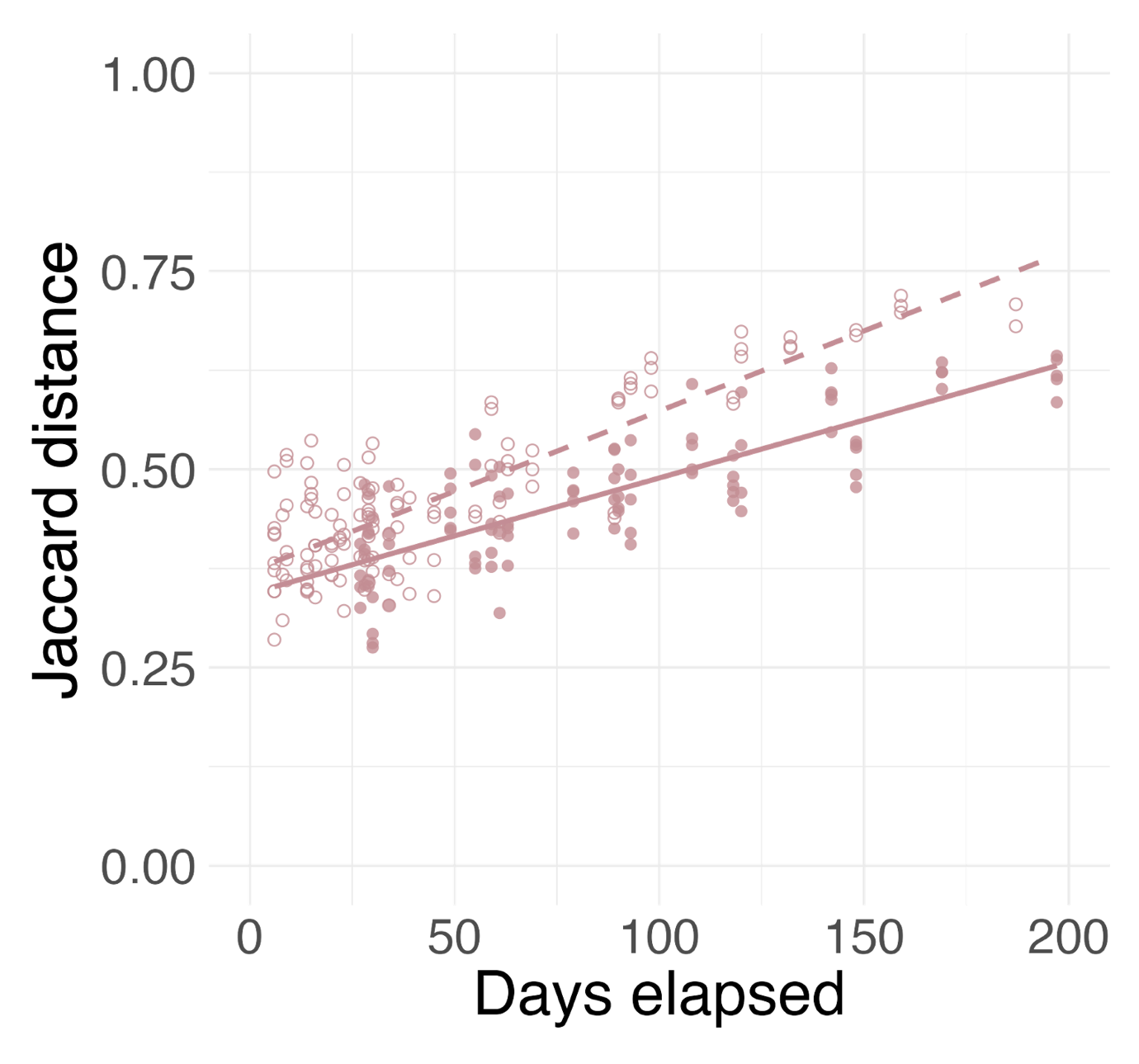


**Supplementary Figure 6.** Within-individual microbiota distance (Jaccard distance) among laboratory mice, according to cage density. Cage density was either three (*empty circles;* linear model, *R^2^=*0.718, *F*_1,125_=322.2, *p*<0.001; *n*=43; 4–6 samples from 9 animals housed across five cages) or five (*filled circles*; linear model, *R^2^=*0.669, *F*_1,97_=198.6, *p*<0.001; *n*=29; 5–6 samples from 5 animals housed in a single cage). All samples were collected from C57BL/6 colony from Animal Facility B.

**
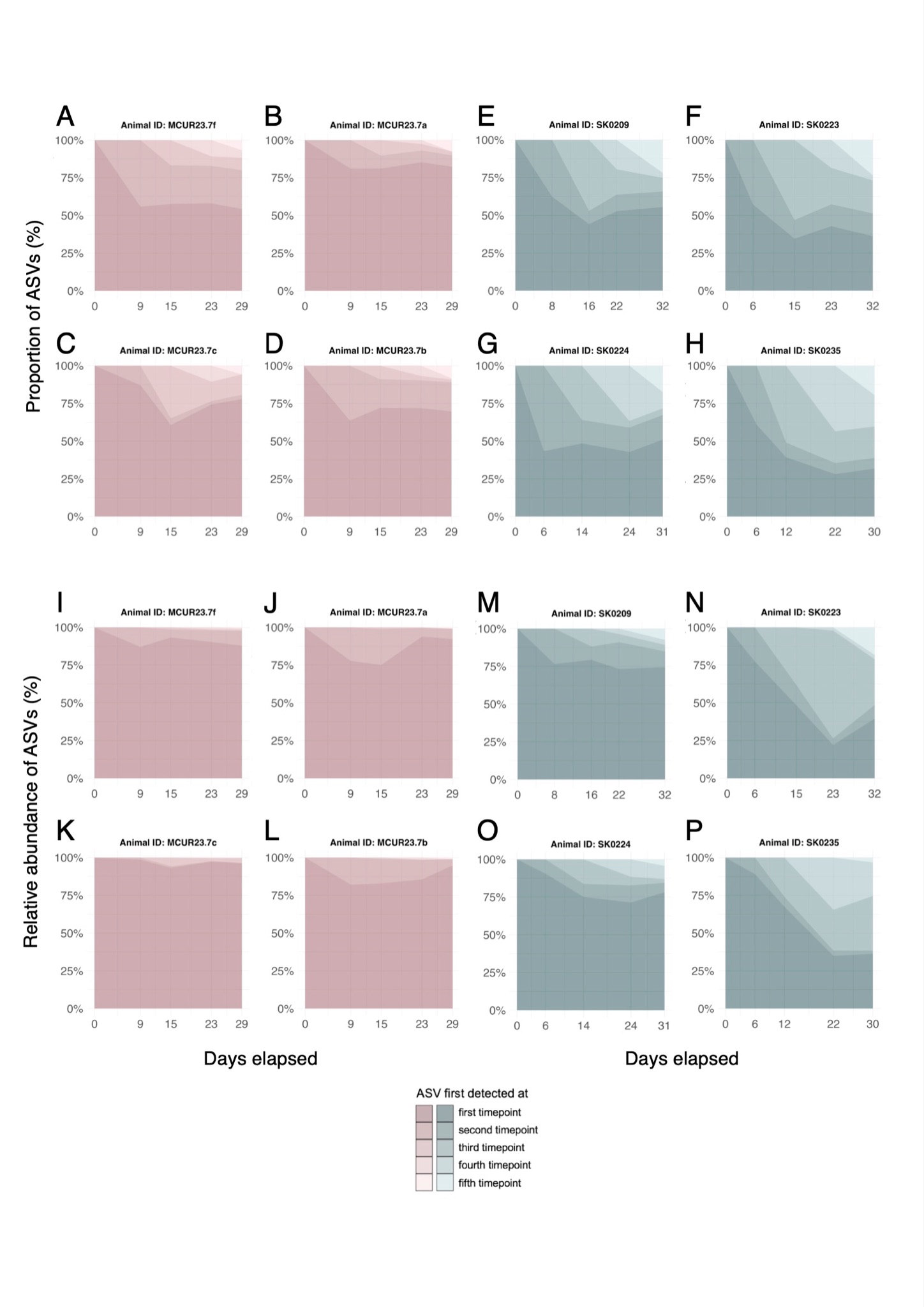
**

**Supplementary Figure 7.** Gut microbiota turnover in adult laboratory (*red*) and wild (*green*) mice. (**A**–**H**) Proportion and (**I–P**) relative abundance of amplicon sequence variants (ASVs) at five timepoints in (**A–D**, **I–L**) laboratory (*n*=5) and (**E–H**, **M–P**) wild mice (*n*=5) mice based on the timepoint the ASV was first detected. Exact sampling points for each animal are presented on x-axes.


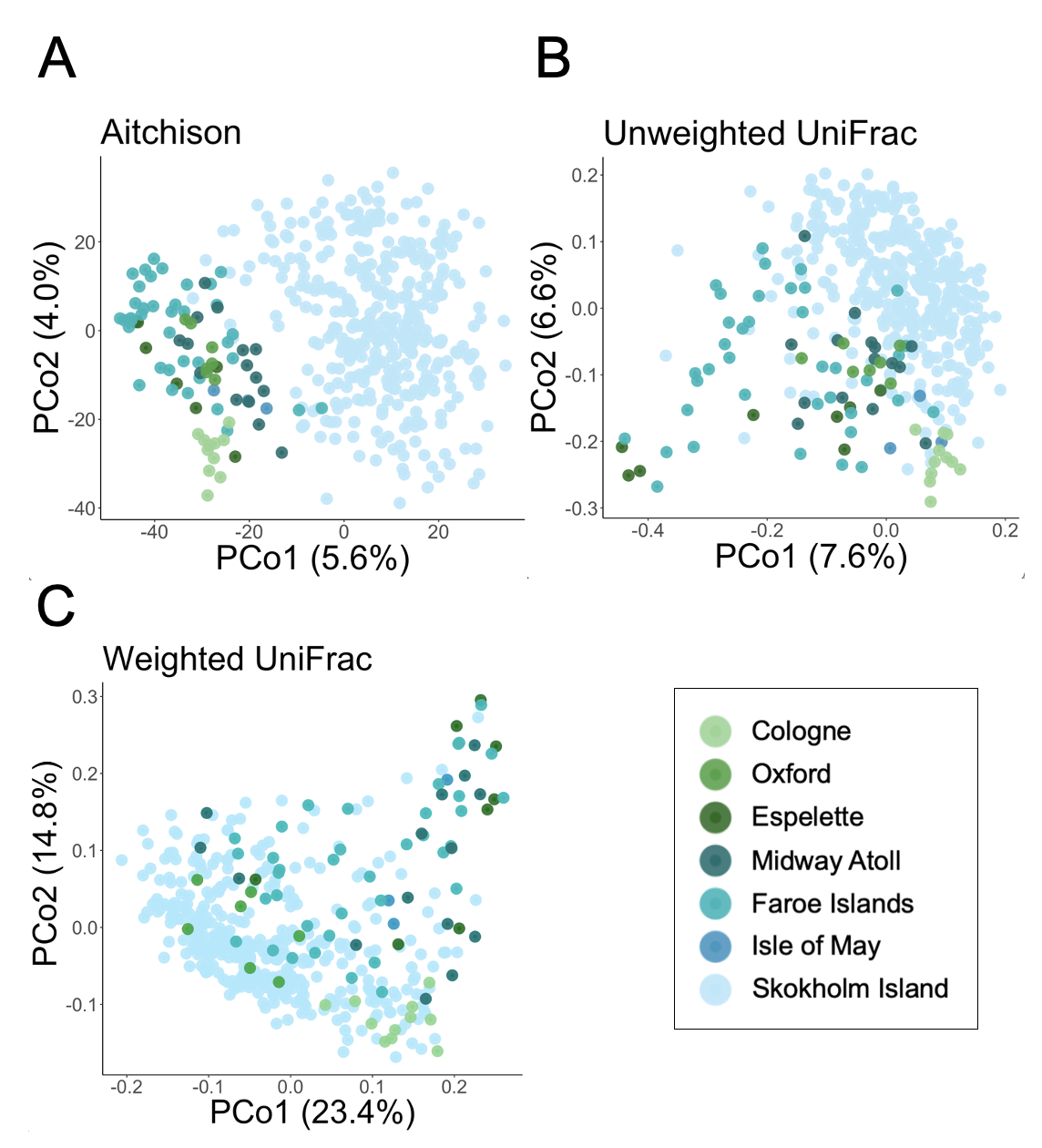


**Supplementary Figure 8.** Principal coordinate analysis of mainland (*green*) and island (*blue*) mouse samples on (**A**) Aitchison, (**B**) unweighted UniFrac and (**C**) weighted UniFrac distances.


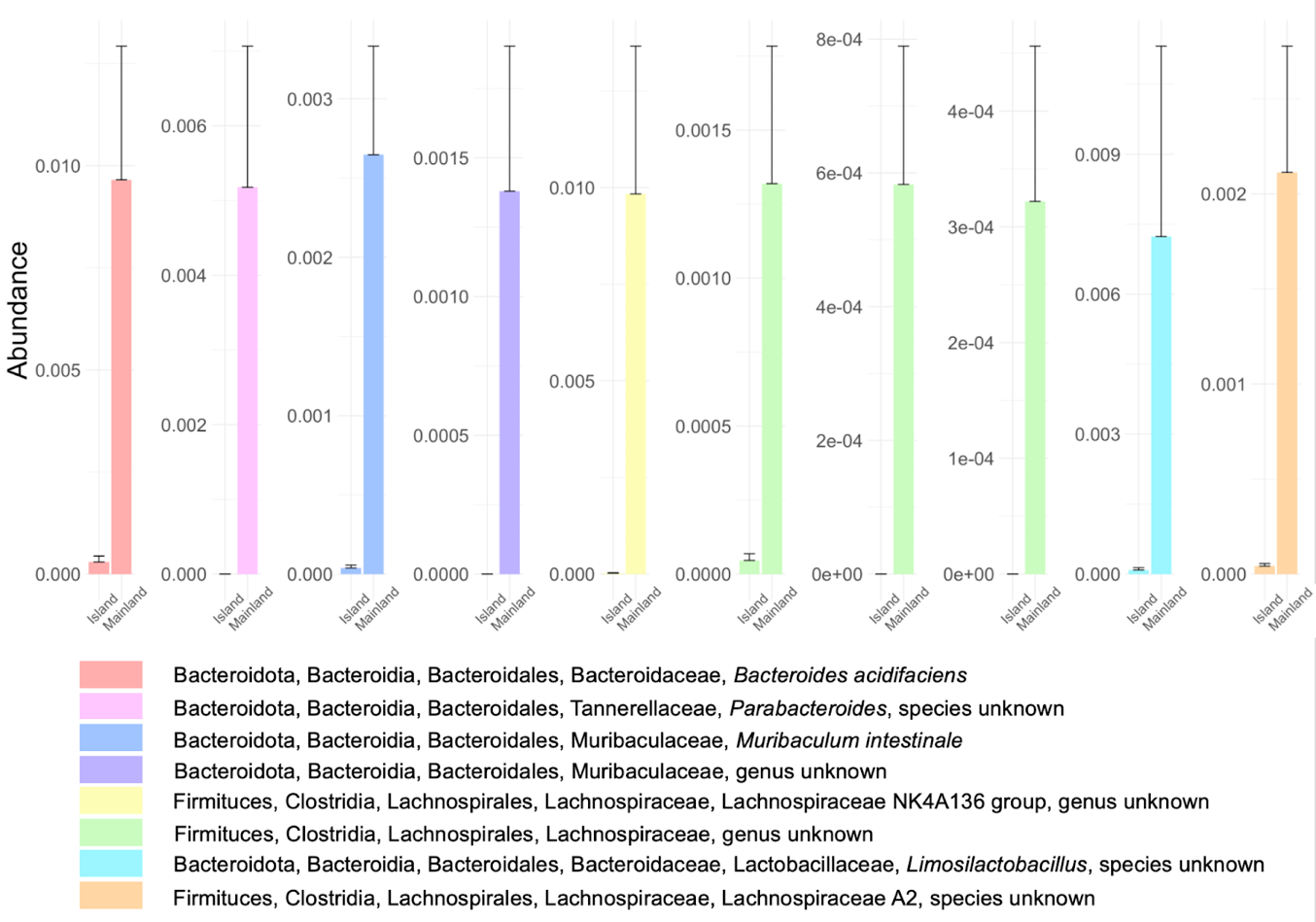
**Supplementary Figure 9.** Relative abundance of top ten amplicon sequence variants (ASVs) predicting setting (island/mainland), ordered in decreasing order of importance from left to right. ASVs were identified with Random Forest regressions, with mean decrease in Gini used as a measure of importance (the higher the score, the higher the importance; OOB estimate of error = 3.9).


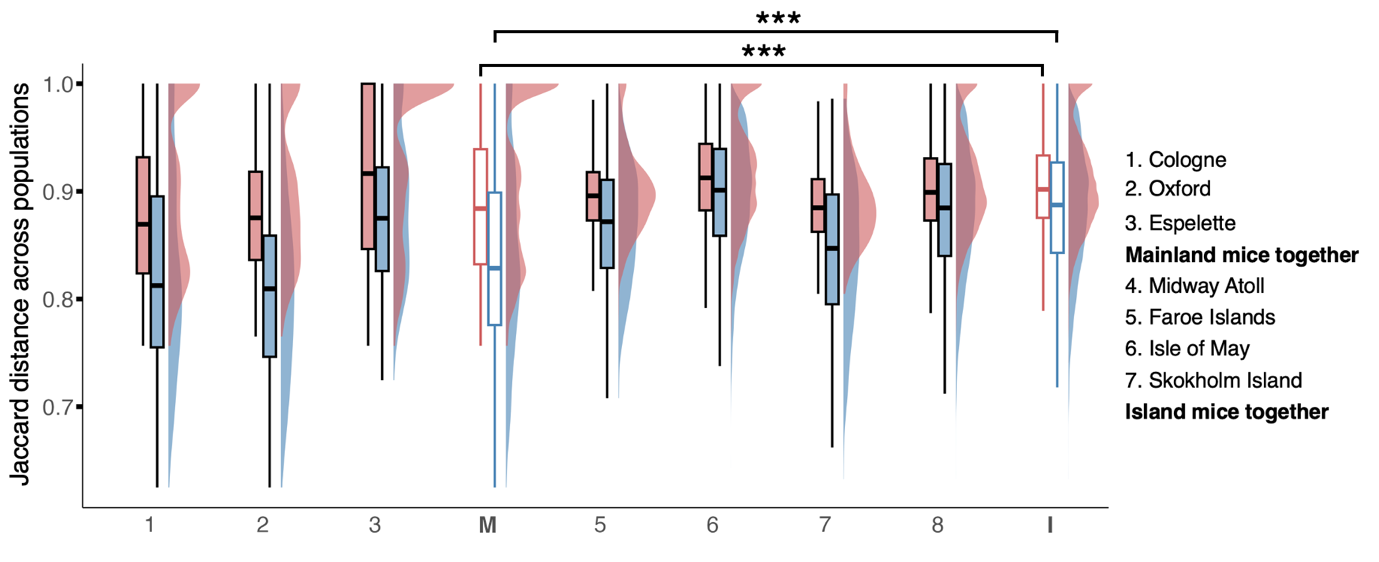


**Supplementary Figure 10.** Pairwise Jaccard dissimilarity of anaerobic (*red*) and aerotolerant (*blue*) gut microbial communities between samples from different mainland or island populations. Statistical differences between mainland and island mice were tested with permutational Wilcoxon rank sum tests (***; *p*<0.001).
